# The impact of 9-azaglycophymine and phenylguanidine derivatives on the proliferation of various breast cancer cell lines in vitro and in vivo

**DOI:** 10.1038/s41598-024-71624-8

**Published:** 2024-11-15

**Authors:** Ibrahim Morgan, Robert Rennert, Robert Berger, Sanja Jelača, Danijela Maksimović-Ivanić, Duško Dunđerović, Sanja Mijatović, Goran N. Kaluđerović, Ludger A. Wessjohann

**Affiliations:** 1 Department of Bioorganic Chemistry, Leibniz Institute of Plant Biochemistry, Weinberg 3, 06120 Halle (Saale), Germany; 2 Department of Immunology, Institute for Biological Research “Siniša Stanković” – National Institute of the Republic of Serbia (IBISS), University of Belgrade, Bulevar Despota Stefana 142, 11108 Belgrade, Serbia; 3 Institute of Pathology, Faculty of Medicine, University of Belgrade, Dr Subotića 1, 11000 Belgrade, Serbia; 4 Department of Engineering and Natural Sciences, University of Applied Sciences Merseburg, Eberhard-Leibnitz-Straße 2, 06217 Merseburg, Germany; 5 Present Address: Berlin, Germany

**Keywords:** Quinazoline, Quinazolinone, Anticancer, Apoptosis, Breast cancer, Structure-activity-relationship, Breast cancer, Cancer therapy, Drug development, Phenotypic screening, Chemical libraries, Drug discovery and development, Screening, Drug screening, Medicinal chemistry, Drug discovery

## Abstract

Quinazolinones, particularly 9-azaglycophymines, and closely related derivatives and precursors were tested in vitro against various breast cancer cell lines representing the major types of breast tumors. Among the 49 compounds tested, azaglycophymine derivative **19** with an electron-withdrawing substituent demonstrated the most significant anti-proliferative effects, with IC_50_ values of around 4 µM. Extensive cell-based investigations revealed that compound **19** induced caspase-dependent apoptosis in HCC1937 (human TNBC), BT-474 (human HER2+/HR+), and 4T1 (mouse TNBC) cells. In contrast, in MDA-MB-468 (human TNBC) and MCF-7 (human HR+) cells, the cell death was induced via a non-apoptotic pathway. The in vivo efficacy of compound **19** was validated using a syngeneic orthotopic 4T1 model in BALB/c mice, resulting in significant reduction of 4T1 breast tumor growth upon intraperitoneal (i.p.) application of doses of 5 or 20 mg/kg. These findings highlight the potential of compound **19** as a promising scaffold for the development of new therapeutic agents for various types of breast cancer and a first structure-activity insight.

## Introduction

The precise regulation of the cellular growth is a tightly controlled process. Conversely, dysregulations may lead to uncontrolled and abnormal proliferation of cells developing a benign or malignant tumor^1^. Amongst the malignant tumor diseases, breast cancer is one of the most often diagnosed types worldwide. In 2022, around 2.3 million cases were newly diagnosed with breast cancer. Breast cancer now accounts for 11.6% of all reported tumors in women and men. Whereby, in women, breast cancers represented 23.8% of all newly diagnosed cancer cases^2^. One of the criteria for the selection of the treatment regimen for breast cancers is the type of receptors overexpressed by the tumor cells. Estrogen receptors (ER) are expressed by around 70–75% of all breast cancer cells, 50% of these cells overexpress additionally the progesterone receptor (PR)^3^. These so called hormone receptor (HR) positive breast cancers can be treated, for instance, with estrogen receptor modulators, such as tamoxifen^4^, or aromatase inhibitors like anastrozole^5,6^. The human epidermal growth factor receptor 2 (EGFR2 or HER2/neu) is overexpressed by around 15–20% of all breast cancer cells^3^. Compared to HR-positive breast cancers, these tumors are in most cases more aggressive and less responsive to conventional chemotherapeutic treatments. However, they can be treated by applying monoclonal antibodies targeting HER2/neu receptors, such as trastuzumab (Herceptin™) or the corresponding antibody–drug conjugates (e.g. trastuzumab emtansine, Kadcyla™)^7,8^. Other breast cancers neither overexpress HRs nor HER2/neu. These types of breast cancer are named triple-negative breast cancers (TNBC). Treatment strategies that can be applied in these cases are, for instance, poly(ADP-ribose) polymerase (PARP) inhibitors, such as olaparib, or programmed death-ligand 1 (PD-L1) blockers like atezolizumab^9,10^. However, the treatment of TNBCs is still challenging. With 5 years survival rates of just 77% in case of TNBCs, the prognosis is worse than for HR- and HER2/neu–positive breast cancers (93%)^11^.

**Fig. 1:**
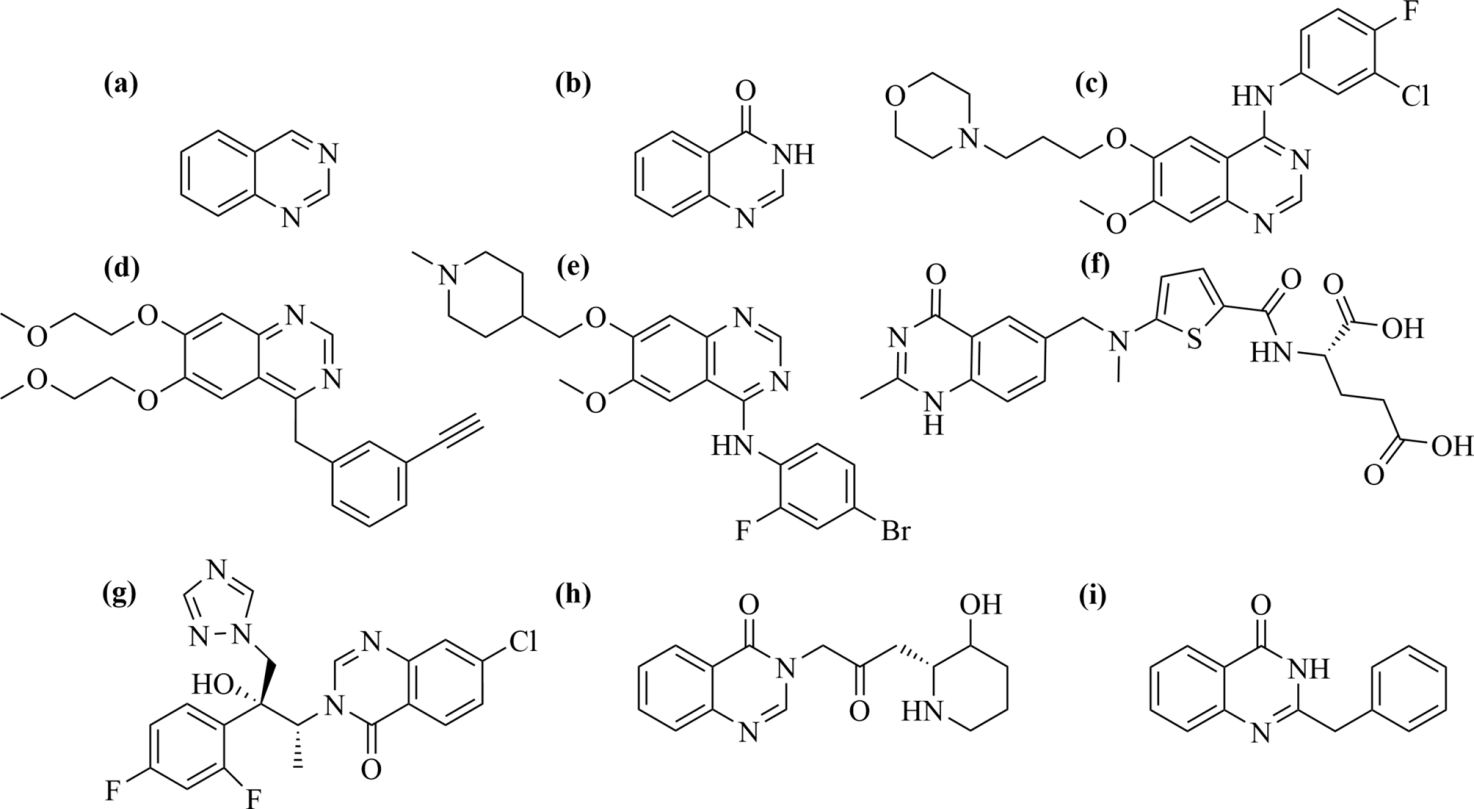
(**a**) Quinazoline, and (**b**) quinazolinone core structures, (**c**) gefitinib, (**d**) erlotinib, (**e**) vandetanib, and (**f**) raltitrexed, (**g**) albaconazole, (**h**) febrifugine and (**i**) glycophymine.

Quinazoline and quinazolinone (illustrated in Fig. 1a and 1b, respectively) derivatives are core structures associated with several pharmaceutical effects. Derivatives thereof have been described to be beneficial as anti-inflammatory drugs by inhibiting NF-κB (nuclear factor kappa-light-chain-enhancer) transcription and TNF-α (tumor necrosis factor alpha) production^12^. Moreover, such compounds have been described to have anti-fungal activities, e.g. albaconazole (Fig. 1g), by inhibiting the cytochrome P450-dependent lanosterol 14α-demethylase (CYP51A1) leading to an inhibition of ergosterol synthesis^13^. Furthermore, several quinazolinone derivatives, e.g. febrifugine (Fig. 1h) isolated from *Dichroa febrifuga*, have been investigated for their anti-malarial effects against *Plasmodium falciparum* and *Plasmodium vivax*^14^.

Other derivatives have been proven to possess anticancer activities and are used as chemotherapeutic agents. Gefitinib and erlotinib (Fig. 1c,d) are quinazoline derivatives which act as inhibitors of epidermal growth factor receptors (EGFRs). They are clinically approved to treat EGFR overexpressing cancers, such as non-small cell lung cancer^15,16^. Vandetanib (Fig. 1e), another quinazoline derivative, is used as chemotherapeutic agent based on its antagonistic effect against the vascular endothelial growth factor receptor (VEGFR) for the treatment of advanced and metastatic thyroid cancers^17^. Another quinazolinone derivative, raltitrexed (Fig. 1f), has a chemical resemblance to folic acid. This similarity causes an anti-metabolite effect and, therefore, an inhibition of the thymidylate synthase and DNA repair. Hence, raltitrexed is used in the treatment of advanced colorectal cancers^18^. Furthermore, these derivatives were found to possess significant potency to inhibit protein kinases which control crucial pathways regulating cell division and thus cancer progression^19^.

Glycophymine (2-benzylquinazolin-4(1*H*)-one, illustrated in Fig. 1i) was originally isolated from *Glycosmis arborea* (Rutaceae, the citrus plant family), and later other species^20^. Several glycophymine derivatives were proven to act as cytotoxins. For instance, 2-(3,4,5-trimethoxybenzoyl)quinazolin-4(3*H*)-one was reported with an IC_50_ (half maximal inhibitory concentration) of 1.22 ± 0.12 µM against the HepG2 cell line^21^. The goal of this study was to investigate potential activities of 2-(phenylamino)quinazolinone (9-azaglycophymine) derivatives, available from previous synthetic works from the compound library of the Leibniz Institute of Plant Biochemistry, against various breast cancer cell lines representing the different overexpression patterns of the breast cancer-related receptor classes. Compound **19** was studied in more detail to determine its biological impact on cell cycle regulation, cellular proliferation, autophagy and apoptosis induction, NO (nitric oxide) production and the formation of ROS (reactive oxygen species). Moreover, the in vivo antitumor effect of compound **19** was proven by using a syngeneic mouse breast cancer (TNBC) model induced by orthotopic implantation of 4T1 cells in BALB/c mice.

## Materials and methods

### Cell lines and chemicals

The breast cancer cells used in this study were the human cell lines MCF-7, BT-474, HCC1937, MDA-MB-468, and the mouse cell line 4T1. The cell culture reagents, RPMI1640, fetal calf serum (FCS), penicillin (10^7^ units/L)/streptomycin (10 g/L) (P/S), Dulbecco's phosphate-buffered saline (PBS), L-glutamine (200 mM), and 0.05% trypsin–EDTA were purchased from Capricorn Scientific (Ebsdorfergrund, Germany). The Endopan 3 medium kit was purchased from PAN-Biotech (Aidenbach, Germany).

Annexin V/propidium iodide (AnnV/PI) kit and trypan blue were obtained from Invitrogen (Waltham, Massachusetts, USA). ApoStat was supplied by R&D systems (Minneapolis, Minnesota, USA). Acridine orange (AO) and crystal violet (CV) were from Sigma Aldrich (St. Louis, Missouri, USA). Carboxyfluorescein succinimidyl ester (CFSE) and dihydrorhodamine 123 (DHR) were purchased from BD Horizon (Franklin Lakes, New Jersey, USA). 4-Amino-5-methylamino-2′,7′-difluorofluorescein diacetate (DAF-FM) was bought from Cayman Chemical Compounds (Ann Arbor, Michigan, USA). Dimethyl sulfoxide (DMSO) was from Duchefa Biochemie (Haarlem, The Netherlands). Acetic acid and paraformaldehyde (PFA) were purchased from Carl Roth (Karlsruhe, Germany), and 4′,6-diamidino-2-phenylindole (DAPI) from Roche (Basel, Switzerland).

### Cell culture

In this study, four human breast cancer cell lines differing in their ER, PR, EGFR, HER2/neu and BRCA-1 (breast cancer gene 1) expression levels were investigated^5^, namely MCF-7, BT-474, HCC1937 and MDA-MB-468. The characteristic expression levels of the essential receptor genes in these human cell lines are illustrated in Table S1^22–24^.

The cell lines were cultured in standard growth medium consisting of RPMI1640 medium supplemented with 10% (v/v) FCS, 1% (v/v) penicillin/streptomycin and 1% (v/v) glutamine^25–29^. Two additional cell lines were included in the study representing non-tumorigenic (“healthy”) breast cells, namely 184B5 and MCF10A. These cell lines were cultured using an Endopan 3 medium kit in accordance with the manufacturer’s instruction. For passaging and seeding, cells were treated as previously described^30^. In brief, seeding was done in low density with 6 × 10^3^ cells/100 µL in 96-well plates, and with 1.5 × 10^5^ cells/1 mL in 6-well plates. Prepared cell flasks and plates were maintained under humidified atmosphere with 5% CO_2_ at 37 °C (standard culture conditions).

### Cell viability assays

For initial testing of the cytotoxic effect of the available glycophymine compounds (Table S2), cells were treated with compound in three concentrations: 1, 10 and 50 µM. Cells were seeded in 96-well plates and allowed to adhere overnight. Then they were treated for 72 h under standard culture conditions. Those compounds that showed a cytotoxic effect when applying ≤ 10 µM concentration and causing a reduction of the cell viability by at least 50% were selected for the determination of their IC_50_ value. For IC_50_ determination, the compounds were tested at seven concentrations (0.78, 1.56, 3.13, 6.25, 12.5, 25 and 50 µM). Subsequently, the cell viability was determined by using the crystal violet (CV) assay. Moreover, the selected compounds were tested against the mouse breast cancer cell line 4T1 (TNBC) as well. The compounds were prepared initially to obtain a stock solution of 20 mM in DMSO. Later, the working solutions were prepared by mixing the required amount of DMSO stock with the optimal medium for each cell line. The DMSO concentration in the final solution was always maintained lower than 0.5% to avoid any DMSO-derived side effects.

For CV assays, after 72 h treatment, the treatment medium was discarded from the 96-well plates, and the cells were washed using 50 µL of PBS. Cells were fixed by adding 50 µL of 4% (v/v) paraformaldehyde (PFA) in PBS for 15 min at room temperature (RT). Afterwards, PFA was discarded, and the plates were dried for 15 min at RT. 50 µL of CV working solution (0.1% w/v CV in PBS) were added to the wells, followed by 15 min incubation at RT. Subsequently, the CV solution was discarded, and the plates were washed using aqua bidest. (ddH_2_O) to remove any remaining CV solution. The plates were left to dry overnight at RT, and then 50 µL of 33% (v/v) acetic acid in ddH_2_O were added to each well. Finally, the absorbance was measured at 570 nm by using a SpectraMax M5 plate reader (Molecular Devices, California, USA) and at 670 nm as background wavelength^31^. The results were normalized using two controls: a negative control of viable untreated cells, prepared by adding 100 µL of compound-free standard growth medium, and a positive control, achieved by treating cells with the cytotoxic saponin digitonin (100 µL solution in standard growth medium; 125 µM final concentration). Additionally, gefitinib, an approved EGFR inhibitor of the quinazoline family, was tested as anti-proliferative positive control^32^. The data represent the mean ± standard deviation derived from three independent biological replicates. Dose–response curves were generated by four-parametric non-linear regression analysis using the GraphPad Prism software (version: 10.1, San Diego, CA, USA). Based on that, IC_50_ values and standard deviations were calculated for each replicate individually.

### Flow cytometric analyses

#### Cell cycle, apoptosis, pan-caspase activation and autophagy analyses

Cells were seeded in 6-well plates and incubated for 24 h under standard culture conditions. Afterwards, the cells were treated with compound **19** at IC_50_ for 72 h under standard culture conditions.

For cell cycle analyses, cell fixation was performed with 70% (v/v) ethanol for 24 h at 4 °C. The cell suspension was centrifuged for 3 min at 800 rpm, and the cell pellet was stained by using DAPI staining solution (1 µg/mL of DAPI, 1% (v/v) Triton™ X-100 in PBS) for 10 min at RT.

For apoptosis analyses, the cell suspension was centrifuged for 3 min at 800 rpm, and the cell pellet was stained by using 100 µL of AnnV/PI working solution (5 µL of AnnV, 2 µL of PI in 100 µL of PBS) for 15 min at RT and protected from light. The staining was terminated by the addition of 900 µL of annexin binding buffer.

For the analyses of pan-caspase activation, the cell suspension was centrifuged for 3 min at 800 rpm, and the cell pellet was stained with 100 µL of ApoStat working solution (1 µL of ApoStat and 5% (v/v) FCS in 100 µL of PBS). Cells were incubated for 30 min under standard culture conditions. The cell suspension was centrifuged for 3 min at 800 rpm, and the cell pellet was washed with 1 mL of fresh PBS.

For the analyses of autophagy induction, the cell suspension was centrifuged for 3 min at 800 rpm, and the cell pellet was stained with 1 mL of AO working solution (1 µg/mL of AO in growth medium). Cells were incubated for 15 min under standard culture conditions. The cell suspension was centrifuged for 3 min at 800 rpm, and the cell pellet was washed with 1 mL of fresh PBS.

Finally, all samples were analyzed by using flow cytometry (FACSAria III, BD Biosciences, New Jersey, USA).

#### Cell division analyses

The cells were stained with CFSE (1 µM) in PBS containing 0.1% (v/v) FCS for 10 min. Afterwards, cells were washed with PBS, and then seeded with growth medium in 6-well plates. The plates were incubated for 24 h under standard culture conditions. After incubation, cells were treated with compound **19** at IC_50_ and incubated for 72 h under standard culture÷ conditions. Subsequently, the cells were detached using 0.5 mL of 0.05% (v/v) Trypsin–EDTA for 3 min at 37 °C. Then trypsin was deactivated by the addition of FCS-containing growth medium. The cell suspensions were collected, centrifuged for 3 min at 800 rpm, and the cell pellets were resuspended in fresh PBS. Finally, the samples were analyzed by using flow cytometry^33^.

#### Investigation of ROS and NO production

For ROS production analyses, the procedure was performed exactly as described above for the cell division experiments, except that the CFSE staining solution was replaced by DHR staining solution resulting in 1 µM of DHR in PBS containing 0.1% (v/v) FCS^34,35^.

For NO production detection, cells were seeded in 6-well plates, allowed to adhere for 24 h under standard culture conditions, then treated with compound **19** at IC_50_, and incubated for 72 h under standard culture conditions. Afterwards, the medium was discarded and the cells were stained with DAF-FM (5 µM) in RPMI1640 with 10% (v/v) FCS for 1 h at 37 °C. The stain was completed by additional incubation for 15 min with serum-free medium. The cells were then detached, re-suspended in PBS and analyzed by flow cytometry.

### In vivo study

An in vivo mouse study was performed according to the guidelines of the European Union and in accordance with ARRIVE guidelines and approved by the Institutional Animal Care and Use Committee at the Institute for Biological Research “Siniša Stanković”—National Institute of the Republic of Serbia, University of Belgrade (323-07-11755/2019-051).

Compound **19** was selected to be tested against a syngeneic breast cancer model induced by orthotopic inoculation of 4T1 mouse breast cancer cells (TNBC) into BALB/c mice. In that exploratory study, we aimed to investigate for the first time a proposed anticancer efficacy of that compound. For that purpose, three groups were included in the study, each comprising eight animals. The resulting total number of 24 animals aimed to avoid under- or overpowering of the study. The animals were kept under standard laboratory conditions with an ad libitum regime for food and water intake. 5 × 10^4^ of 4T1 cells in 50 µL PBS were inoculated in the fourth right mammary fat pad of female mice at day 0. Anesthesia was induced using a combination of ketamine and xylazine at doses of 60 mg/kg and 7.5 mg/kg, respectively.

On day 6, the mice were assigned to the study groups using a stratified randomization approach, ensuring that the mean tumor volumes were as similar as possible across all groups. The control group was treated with 400 µL of vehicle solution, the low dose compound **19** group with 5 mg/kg in 400 µL of vehicle, and the high dose group with 20 mg/mL in 400 µL of vehicle. The vehicle was 4% (v/v) DMSO in PBS, and the administration route was intraperitoneal (i.p.). The animals were treated daily on days 6–10 (starting from the day when tumors became palpable), 13–17 and day 20. Animals were visually examined daily, and urine samples were collected on the first and last day of treatment and analyzed by Multistix urine test stripes (Siemens Healthineers, Erlangen, Germany). On day 21 of the experiment, the mice were sacrificed using cervical dislocation. Following the sacrifice, the tumor diameters were measured using a caliper. The tumor volume (V) was then calculated using the formula V = L*W^2^*0.52^36^, where L represents the longest diameter and W the shortest diameter of the tumor.

### Histopathology

Tissue samples of tumors, kidneys, livers and spleens were fixed in 4% (v/v) formalin. After fixation, tissues were embedded in paraffin blocks, and 4 µm thick sections were obtained, mounted on glass slides, stained with Hematoxylin Eosin, and analyzed by using an Olympus BX43 microscope (Olympus, Tokyo, Japan). All slides were scanned with a Leica Biosystems Aperio AT2 with ×400 magnification (n = 3 per group). Virtual slides generated from Leica Aperio AT2 (Wetzlar, Germany) were additionally analyzed with Aperio ImageScope (v12.4.3.5008) and with FIJI-ImageJ software.

### Statistical analysis

The data in this study are expressed as mean values with standard deviation calculated based on at least three independent biological replicates, unless differentially specified in the figure captions. Significance levels are denoted as follows: * and # indicate adjusted p-values of less than 0.05 and 0.1, respectively, upon comparison to the untreated control group. Statistical analysis was conducted using ordinary two-way analysis of variance (ANOVA) followed by Sidak’s multiple comparisons test with GraphPad Prism software (San Diego, CA, USA).

## Results

### Cell viability assay

**Fig. 2:**
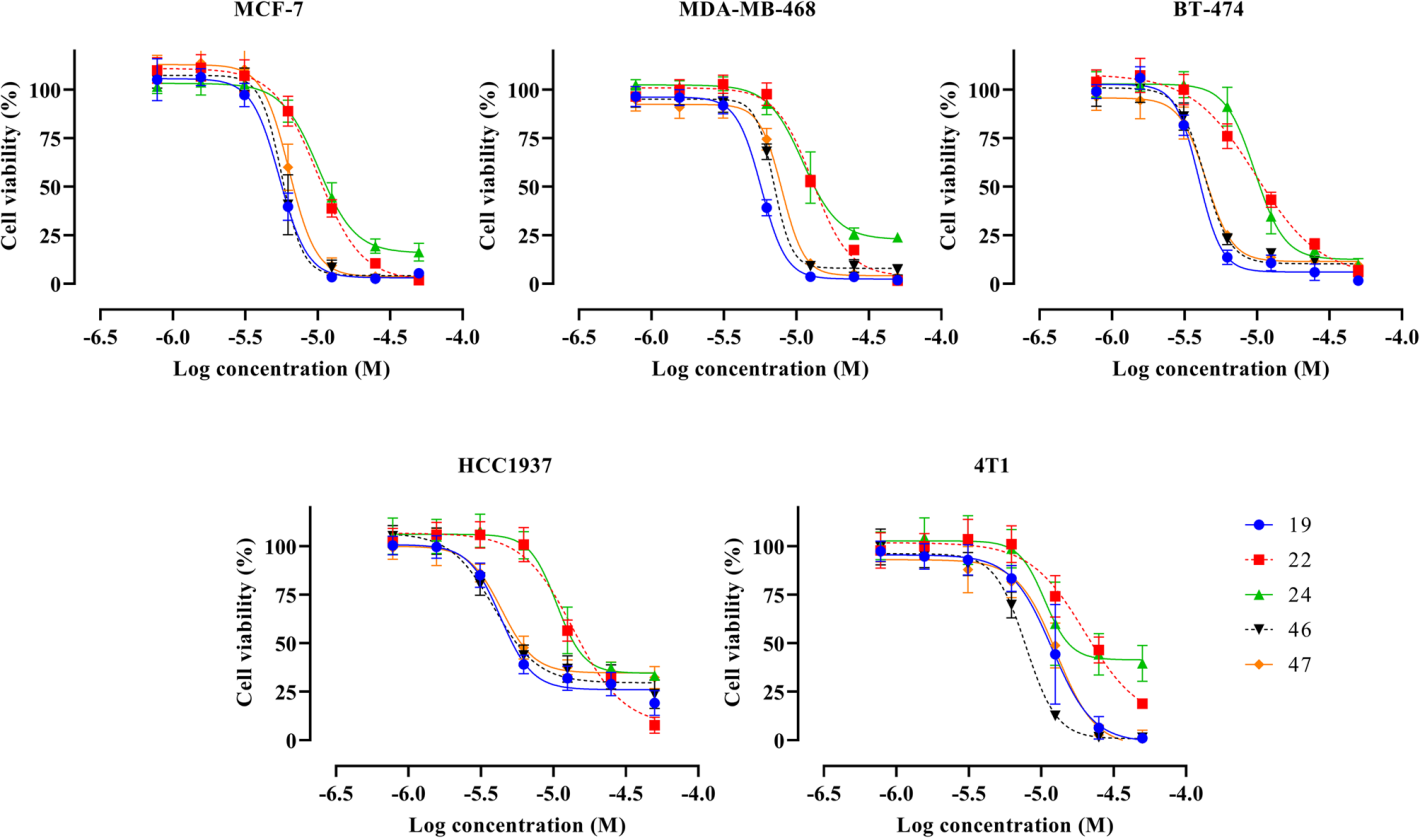
Dose–response curves of the viability of MCF-7, MDA-MB-468, BT-474, HCC1937 and 4T1 cells after 72 h treatment with the most active compounds, measured by using the CV assay.

Fast screening was performed to determine the anti-proliferative activity of 49 compounds (Table S2) against MCF-7, MDA-MB-468, BT-474 and HCC1937 breast cancer cells. The cell viability detected after 72 h of treatment are illustrated in Fig. S1 and S2. The compounds **19**, **22**, **24**, **46** and **47** significantly reduced cell proliferation of all four cell lines. These compounds were selected for further studies, testing them in a wider range of concentrations to determine their IC_50_ values (as a mean), using a four-parameter logistic function. Moreover, the most active compounds were also tested against the aggressive, triple negative 4T1 mouse breast cancer cell line, aimed for the selection of the most relevant substances for further syngeneic in vivo mouse studies. The obtained cell viability dose–response curves are shown in Fig. 2, the accordingly calculated IC_50_ values are summarized in Table 1.

**Table 1:** IC_50_ values (µM) of the most active compounds as determined for the cell lines under investigation.

Compound	MCF-7	MDA-MB-468	BT-474	HCC1937	4T1
**19**	5.5 ± 0.6	5.8 ± 0.3	4.0 ± 0.2	4.3 ± 0.1	12.5 ± 3.7
**22**	10.0 ± 0.5	13.0 ± 0.4	9.9 ± 1.5	14.8 ± 3.7	23.5 ± 7.2
**24**	10.3 ± 1.3	11.2 ± 1.8	9.7 ± 1.3	11.0 ± 1.9	11.3 ± 3.5
**46**	5.7 ± 0.6	6.9 ± 0.2	4.4 ± 0.1	3.9 ± 0.2	7.9 ± 0.6
**47**	6.3 ± 0.4	8.0 ± 0.3	4.5 ± 0.2	4.4 ± 0.3	12.6 ± 1.2

Compound **19** was found to be the most active test item against most of the breast cancer cell lines, with IC_50_ values of 4–6 µM. Although compounds **46** and **47** showed similar results, they were slightly less active in some cell lines. Based on these data, compound **19** was selected for further analyses using flow cytometry and in vivo application.

### Flow cytometry

**Fig. 3:**
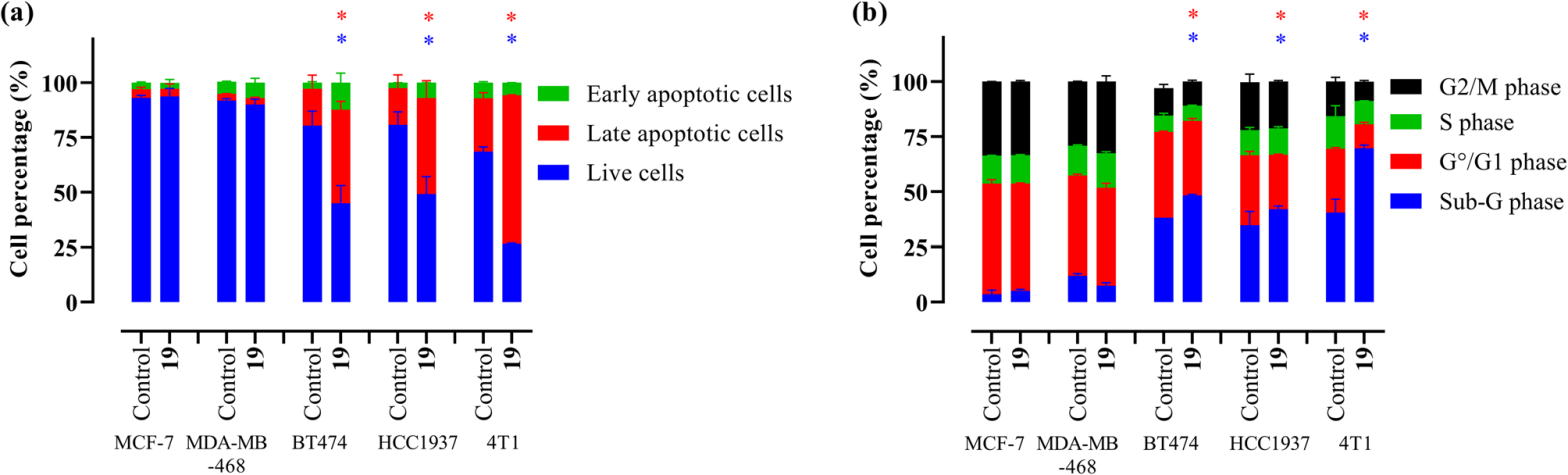
Bar graphs representing the biological impacts of compound **19** applied at IC_50_ for 72 h to several breast cancer cell lines on (**a**) the induction of apoptosis, and (**b**) the cell cycle status distribution in G0/G1, S and G2/M phases. Bars represent the mean values ± standard deviation calculated based on three independent measurements. *p < 0.05 compared to the untreated control cells.

Flow cytometric studies pointed out that compound **19** varies in its modes of action in different cell lines. In BT-474, HCC1937 and 4T1, it induced an inhibition of proliferation and an activation of pan-caspases leading to apoptosis (percentage of apoptotic cells increased upon treatment with **19** when compared to control cells as follows: in treated BT-474 51% versus 20% in control cells, in HCC1937 47% versus 19%, and in 4T1 69% versus 31%, as illustrated in Fig. 3a). The presence of acidic vesicles including autolysosomes, which is an indicator of autophagy activation^37,38^, was not changed by the treatment. Interestingly, enhanced NO production in BT-474, HCC1937, 4T1 and MCF-7 was well synchronized with ROS downregulation and pan-caspase activation, except in caspase-3 deficient MCF-7 cells. This might be caused by inhibition of membrane enzymes which produce superoxide anion by NO, further on enabling pan-caspase activation and successful induction of apoptosis^39^. In this context, MCF-7 cells could not respond to the treatment by induction of the caspase-3 initiated intrinsic apoptosis program, despite excessive production of NO. Interestingly, in MDA-MB-468, the confirmed cytotoxic effect of compound **19** seemingly is not mediated by any of the investigated processes. The functional impacts of compound **19** on the cell lines is summarized in Table 2. The underlying histograms, dot plots and bar graphs are shown in the Supplementary Information Figs. S3–S10.

**Table 2:** Cell line-dependent biological impacts of compound **19** treatment with IC_50_ for 72 h. ↑—induction; ↓—inhibition; ↔—no effect. The bar graphs used to determine these data are illustrated in Fig. S10.

Cell response	MCF-7	MDA-MB-468	BT-474	HCC1937	4T1
ROS	↔	↔	↓	↓	↔
NO	↑	↔	↑	↑	↑
Autophagy	↔	↔	↔	↔	↔
Proliferation	↔	↔	↓	↓	↓
Pan-caspases	↔	↔	↑	↑	↑

### In vivo study

**Fig. 4:**
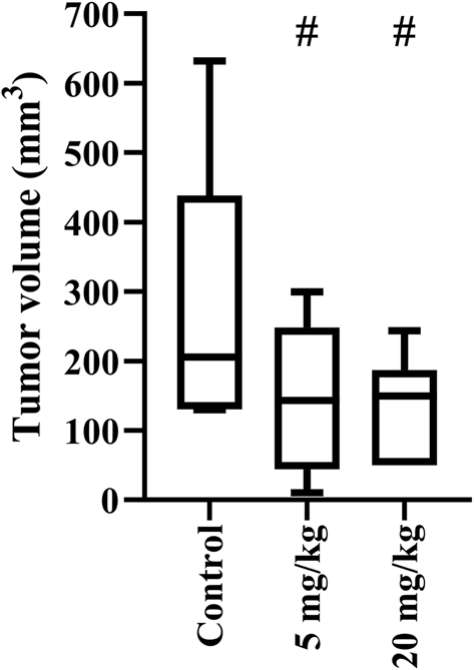
Impact of compound **19** on the 4T1 (mouse breast cancer cell line) tumor progression in a BALB/c mouse orthotopic model using two different doses, 5 and 20 mg/kg, applied once daily via i.p. route. Compound **19** was applied on days 6–10, 13–17 and day 20, whereby day 0 was the inoculation day. The mice were dissected on day 21, and the tumor volumes were measured. #*p* < 0.1 compared to the untreated control group using a caliper.

The i.p. treatment with 5 mg/kg of compound **19**, decreased the median tumor volume from 205 mm^3^ (control group) to 143 mm^3^ (5 mg/kg treated group) as illustrated in Fig. 4. Using a higher dose of 20 mg/kg, no further improvement of the tumor suppression was observed. The systemic toxicity of the treatment was monitored regularly by observing the animals’ body mass (Fig. S11), behavioral changes, fur color and loss, and food intake (data not shown). However, no significant abnormalities of these health parameters were observed during the whole study, indicating the absence of acute systemic toxicity caused by compound **19**. In addition, biochemical markers measured in urine samples indicated compound **19**’s lack of systemic toxicity for both doses tested (Table S3).

### Histopathology

**Fig. 5:**
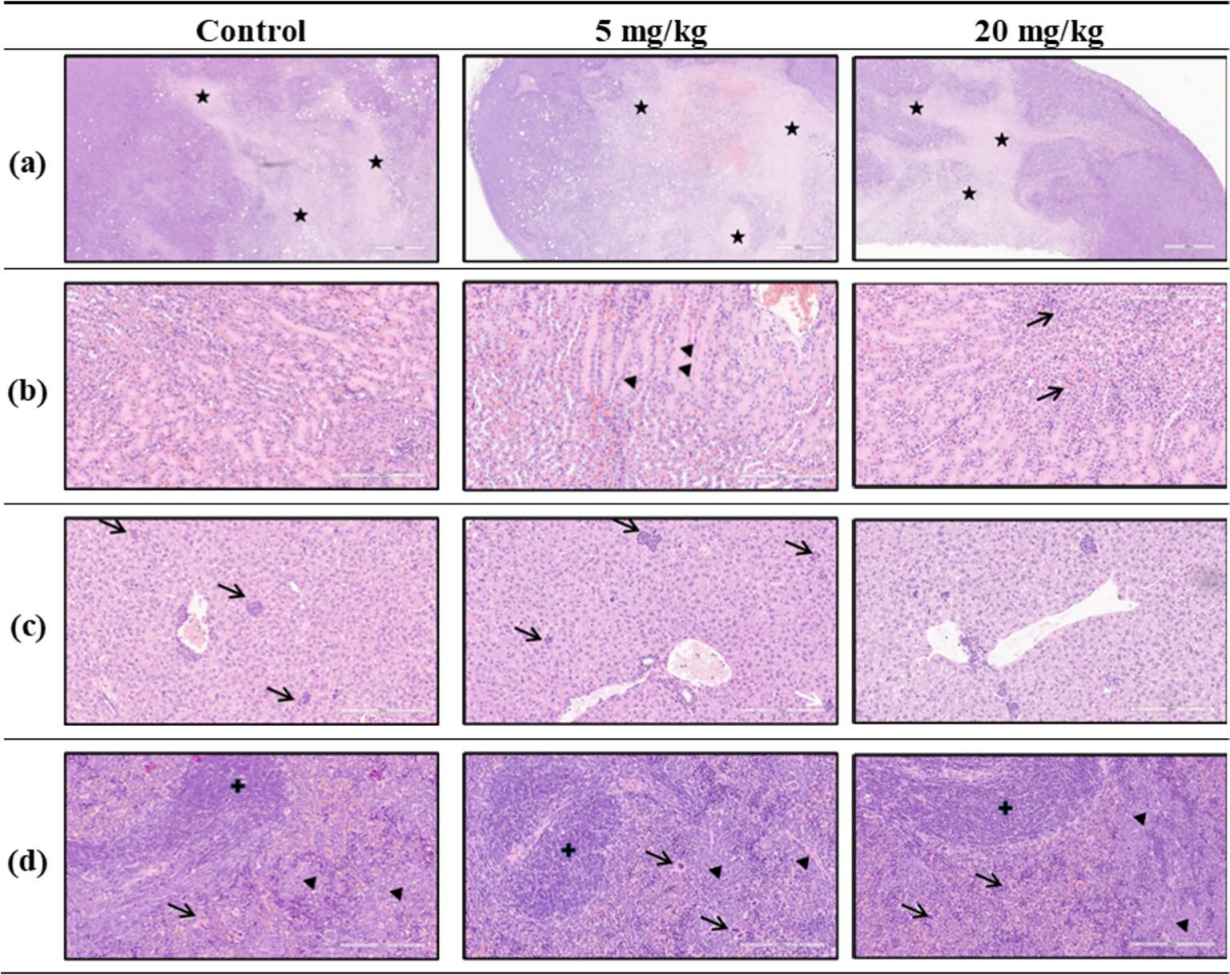
Histopathological assessment. BALB/c mice were inoculated with 4T1 cells at day 0, eight animals per group were treated with vehicle, 5 mg/kg or 20 mg/kg, respectively. The dissection and the organ isolation were performed on day 21. (**a**) Tumor tissues with necrotic areas (black stars) (H/E stain, ×40). (**b**) Kidney tissues with intratubular protein casts (black arrowheads) and lymphocytes in interstitial space (black arrows) (H/E stain, ×200), respectively. (**c**) Liver tissues with intrasinusoidal extramedullary hematopoiesis (black arrows) (H/E stain, ×200). (**d**) Extramedullary hematopoiesis with megakaryocytes (black arrows) and hematopoietic cells (black arrowheads) in red pulp of spleen (white pulp is labeled with black cross) (H/E stain, ×200).

Along with the aforementioned efficacy study, tissue samples of the 4T1 breast tumors, kidneys, livers and spleens were taken from all mice and analyzed histopathologically (Fig. 5). Necrotic sections were present in all tumor tissue samples including the samples of the control group, except for one sample treated with 20 mg/kg of compound **19**. In that specific animal, the tumor volume was the lowest in the group and stayed without necrotic core. The extent of necrosis was variable, ranging from the absence of necrosis to around 85% of tumor tissue being necrotic in the cross section. However, in comparison to the control group, a higher extent of necrosis was found in treated animals. Kidneys from the control group showed no changes. Furthermore, some additional parameters were observed to be slightly changed in both dosing groups. Those changes were represented by the presence of tubular protein casts, foci of periglomerular fibroblastic proliferation, and paucicellular interstitial medullary lymphocytic infiltrates. Extramedullary hematopoiesis was present in all samples of liver and spleen tissues, independent of vehicle or compound treatment, as it is described in the same tumor model by Tao et al.^40^, postulating that the induction of hematopoiesis in the liver and spleen was induced rather by the tumor cells themselves, than by the treatment. Hematopoietic cells in the liver were present in small groups within liver sinusoids. The spleen's red pulp was occupied by hematopoietic cells, with easily visible megakaryocytes. In conclusion, histopathological inspections showed that compound **19** treatments did not cause significant morphological changes in the kidney, spleen and liver tissue when compared to the control group, indicating the absence of substantial acute tissue toxicity.

## Discussion

**Fig. 6:**
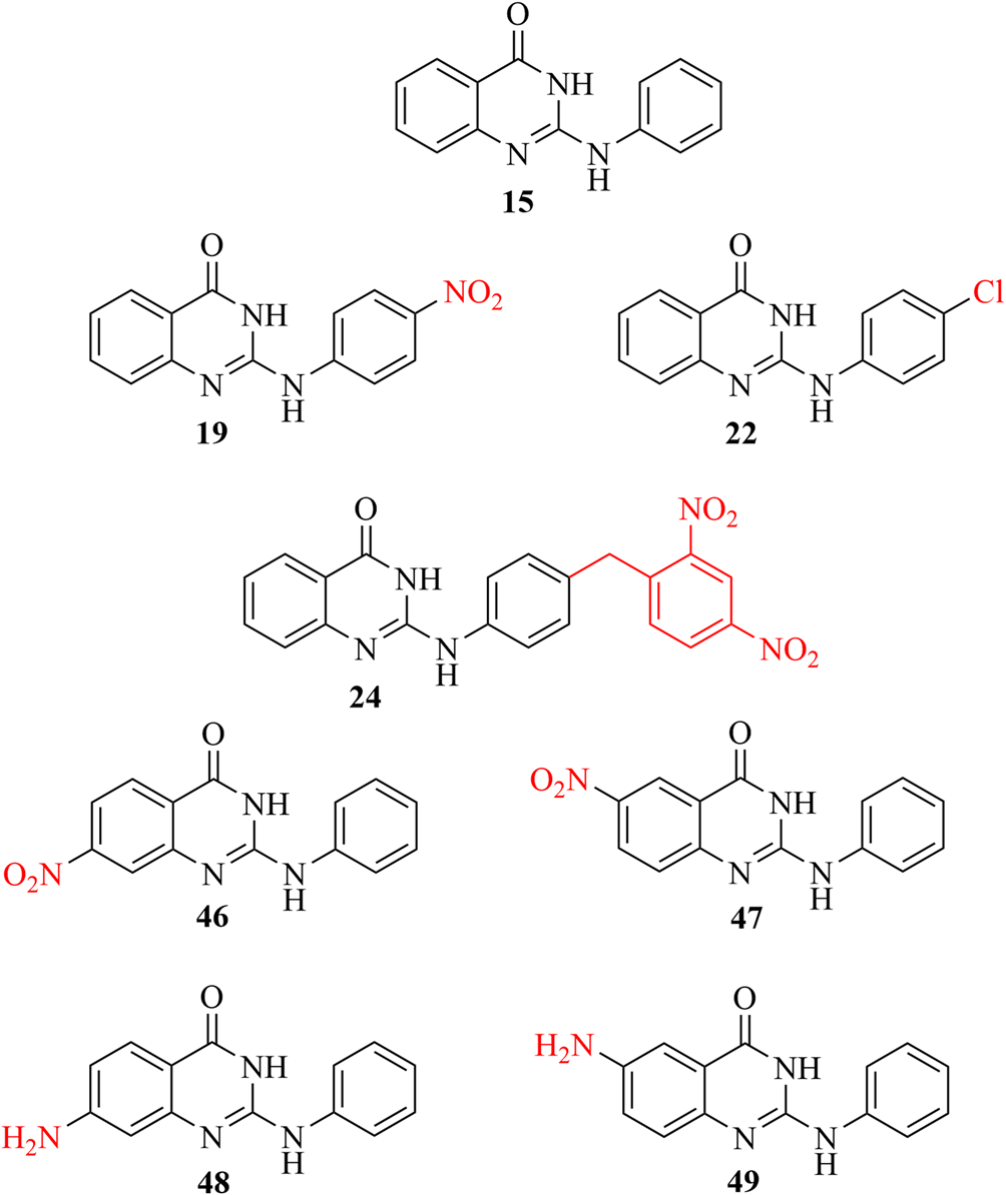
Structures of compound **15** (2-(phenylamino)quinazolin-4(1*H*)-one, 9-azaglycophymine) and the anti-proliferative compounds **19, 22, 24, 46, 47, 48** and **49**. The complete set of compounds **1**–**49** is shown in Table S2.

In this study, the biological effects of several derivatives of 2-(phenylamino)quinazolin-4-one (Fig. 6, parent compound **15**) on various breast cancer cell lines were extensively studied. Out of initially 49 screened compounds, five hits (**19**, **22**, **24**, **46** and **47**) caused ≥ 50% reduction of cell viability after 72 h of treatment, recognizing them as bioactive compounds based on the National Cancer Institute guidelines^41^, as they possess IC_50_ values < 30 µg/mL. All these compounds contain nitro- or amino-substituents at the aromatic benzene rings, however, the nitro compounds clearly being the more active ones with similar IC_50_ values in their group, interestingly, independent of the positioning of the nitro group. Compound **19** was slightly more active against most of the breast cancer cell lines under investigation, especially against BT-474 with an IC_50_ of 4 µM.

Several structural features have been noticed to contribute to the cytotoxicity of quinazolinones. The core structure without any modifications did not show any cytotoxic effect against the breast cancer cell lines up to 50 µM (compound **15**, Fig. S1, S2). However, substituents at the core structure (compounds **19**, **22**, **24**, **46** and **47**), drastically increased its cytotoxicity. The impact of the nitro group (compounds **46** and **47**) becomes obvious when compared with the respective amino group derivatives (compounds **48** and **49**, respectively), which completely lack cytotoxic activity. This is of importance, since aromatic nitro groups (“nitroarenes”) are considered as metabolically instable, being prone to be reduced in its various stages to amino groups^42,43^. However, in medicinal chemistry they also constitute an isostere and substitute for a carboxylate in hydrogen bridging, but with the difference of being strongly electron withdrawing to the aromatic core. Also aromatic amines play a dual role, being indispensable moieties in some drugs while being suspicious of causing cancer in other compounds (hair dye aniline derivatives, for instance), depending on compound structure and application context^44–46^.

Contrarily, the exact position of the nitro group seems to have little effect on the molecule’s anti-proliferative activity. This might be seen as indication that its physicochemical (electron-withdrawing) properties are most relevant, while a function as hydrogen bridge acceptor would be expected to require explicit regioselectivity. Importantly, compound **22**, lacking a nitro group, is cytotoxic as well, albeit twofold less than compound **19** (Fig. 6). What can be discussed as another indication that the electron-withdrawing effect on the core 2-(phenylamino)quinazolin-4(1*H*)-one is crucial for cytotoxic efficacy, not the nitro group as such. However, compound **24**, decoupled from the core, shows a similar effect as **22**.

Compound **19** showed the strongest anti-proliferative and cytotoxic activity. It caused a cell viability reduction in BT-474 cells by 50% after 72 h treatment at a concentration of 4.0 µM. Hence, **19** was selected for more detailed studies in order to determine its mode of action. Indeed, compound **19** triggered various cell-specific responses. In BT-474, HCC1937 and 4T1 cells it caused caspase-dependent apoptosis (Fig. 3, Fig. S3)^47^. This process culminates in the cleavage of chromosomal DNA into oligonucleosomal fragments which explains the accumulation of the hypodiploid cells in the sub G1 phase in the cell cycle assay (Fig. 3, Fig. S9)^48^. It is indicative that in all three cell lines mentioned above enhanced production of NO was orchestrated with diminished ROS generation. One of the possible explanations for this is the fact that in the membrane of these cells, NADPH oxidase (NOX) complexes can actively produce moderate quantities of superoxide anions and provoke tumor progression. NO generated in response to the treatment inhibits their activity, preventing superoxide anion release, thus liberating caspase-3 from O_2_-mediated suppression, resulting in completion of the apoptotic process. Consequently, ROS accumulation was disrupted, leading to a ROS-deficient environment which is not stimulative for tumor cell metabolism and proliferation^39^. Contrarily, in case of MCF-7 and MDA-MB-468, caspase-dependent apoptosis had to be excluded as potential cause for the observed cell death, since compound **19** did neither activate caspases nor apoptotic cell death in these cell lines. Indeed, it was previously described that caspase-3 deficient MCF-7 cells^49^ usually undergo other cell death programs. For instance, it could undergo necroptosis^50^ or entosis^51^. These special cell death processes could be an explanation for compound **19**’s effect on this cell line. However, that has to be investigated in more detail in an advanced future study. Contrarily to the other investigated breast cancer cell lines, MDA-MB-468 is characterized by a tremendous expression of EGFR^52,53^. As the use of gefitinib was described to induce cancer cell death by an activation of caspases-independent pathways in EGFR-expressing cells^54^, one can thus hypothesize that the structurally similar compound **19** might act similarly. Hence, the comparative assessment of compound **19**’s toxicity with gefitinib was conducted on MDA-MB-468. The assay was performed following the previously mentioned viability assay protocol. The results indicated that compound **19** has nearly three times higher toxicity against MDA-MB-468 in comparison to gefitinib, as illustrated in Fig. S12. However, further investigations are essential to eventually prove that the impact of compound **19** on this cell line is causally mediated through the EGFR pathway. Based on the previously mentioned information, the caspase family might play a crucial function in mediating the distinct modes of action of compound **19** in different cell lines. This asks for additional future studies to deeply explore the impact of **19** or improved derivatives (see discussion) on various caspase pathways. It is noteworthy to mention that during evaluating the effect of the compound on healthy breast cell lines, namely MCF-10A and 184B5, it was noticed that the compound lacks selective cytotoxicity to these. The lack of selectivity could be related to the common tissue of origin for these cell lines, healthy and malignant. Therefore, they share similar biochemical settings and hence they respond similarly but obviously not to other tissues – as noticed in the in vivo studies. The compound’s induced cell death in these cell lines, exhibiting a comparable IC_50_ value of approximately 4 µM, is shown in Fig. S13. Proper targeting or further chemical derivatization of the lead structure **19** is highly recommended to optimize its selectivity index towards cancer cell treatment.

The 4T1 cell line is an established animal model for stage IV human triple-negative breast cancer derived from BALB/c mice, known for its high invasiveness and tumorigenicity^55^ (p. 1). 4T1 cells are challenging as they have the capability to metastasize to various locations such as bone, liver, or lung. Since compound **19** had an in vitro IC_50_ of 12.5 µM (Fig. 2, Table 1), it was tested in vivo with two different doses of 5 and 20 mg/kg against the syngeneic and orthotopic 4T1 cell line-based BALB/c mouse model. At the lower dose, compound **19** provoked 30% reduction of the tumor volume (Fig. 4) compared to the control group. Interestingly, the higher dosage of 20 mg/kg did not further increase the compound’s effectiveness indicating that the effective therapeutic dose has been reached already with 5 mg/kg, or secondary factors, like bioavailability or ADME effects, limited the in vivo effect of the higher dosage. Assessing the shrinkage of the tumor holds paramount importance, yet it is not the sole consideration. Equally vital is evaluating the composition and characteristics of any remaining tumor mass. Therefore, a histopathological analysis was performed. It revealed massive necrosis within the tumor mass after treatment. The observed effect was associated with a reduced tumor volume, indicated by its positive involvement in therapy-induced tumor tissue damaging. The role of necrosis in tumor progression and its role in response to therapy is highly controversial^56^. Many authors have shown that the presence of central necrosis is a poor prognostic marker in numerous tumor types^57^. However, numerous contradictory data about the meaning of the presence of necrotic areas in breast tumor tissue mainly due to its heterogeneity, are available. They range from strong correlation between the presence of necrosis and tumor grade, aggressiveness, and unfavorable outcomes, indicating that even centrally necrotizing breast cancer should be classified as a new type, to those showing that there was no association between necrosis and tumor progression and prognosis^58,59^. In addition, several studies have revealed necrosis as an important pathological predictive factor for therapy response^60^. Given that cancer cells develop numerous mechanisms to avoid the induction of cell death in the first instance of apoptosis, there has been great interest in recent years for agents able to initiate regulated forms of necrosis. In general, spontaneous versus drug-induced necrotic changes often have the opposite effect on tumor progression and disease outcome^56^. In this context, the relevance of our finding of increased tumor necrosis after treatment remains unclear.

Apart from the obvious antitumor effect, no systemic toxicity of the treatment was observed based on the biochemical, behavioral, and external appearance of the animals. Furthermore, minor histopathological changes, dominantly in the kidney, from the histopathologist’s view were rather related to the tumors influence and not to compound **19** treatment, which did not significantly damage the non-tumor tissues. However, the absence of apparent morphological changes visible by microscopy must be correlated with the functional status of the liver and kidneys to exclude ultrastructural changes not microscopically visible, e.g., by conducting urine sample tests. Taken together, the new experimental therapeutic 4′-nitro-9-aza-glycophymine (**19**) shows promising in vitro and in vivo efficacy against both hormone receptor- (MCF-7) and HER2/neu-positive (BT-474) cell lines but also to hormone-independent, more aggressive triple-negative breast cancer types (MDA-MB-468, HCC1937 and 4T1). Other derivatives with electron-withdrawing substituents, ideally such not being metabolically labile like the nitro group, e.g., fluorides, might result in even better azaglycophymine derivatives to be tested in the future.

## Conclusions

**Fig. 7:**
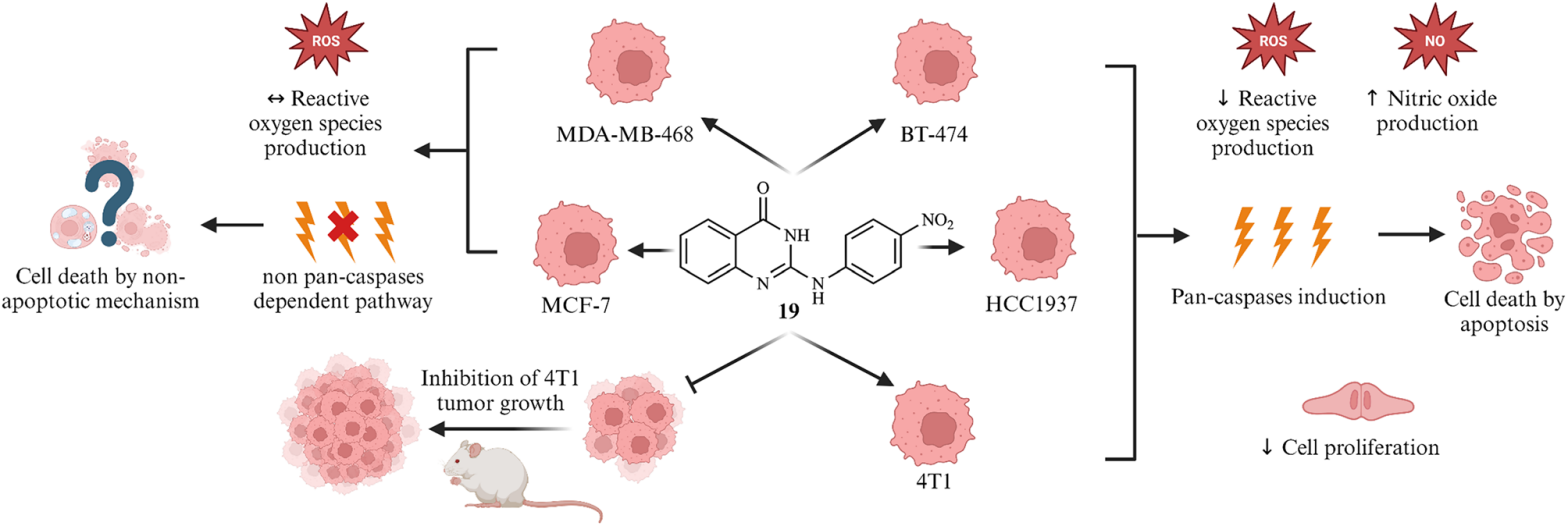
Summary of the modes of cell death induced by compound **19** in the various breast cancer cell lines under investigation (scheme was created by using BioRender.com).

The main goal of the described work was to investigate anticancer activities of 9-azaglycophymine derivatives against various breast cancer cell lines with different genetic background, namely MCF-7, MDA-MB-468, BT-474 and HCC1937, as well as the mouse 4T1 cell line. 49 compounds were initially screened for their cytotoxicity, whereof the compounds **19**, **22**, **24**, **46** and **47** were active below 10 µM concentration. The IC_50_ values of these hit compounds were subsequently determined ranging from 3.9 to 14 µM. Whereby, compound **19** permitted the highest anti-proliferative effects against most of the investigated human breast cancer cell lines with the lowest IC_50_ of 4 µM against HER2/neu-positive BT-474 cells. **19** induced caspase-dependent apoptosis in BT-474, 4T1 and HCC1937 cells. Contrarily, in MCF-7 and MDA-MB-468 cells the compound caused its cytotoxic effect without engagement of any of the investigated cell death processes, indicating that cell line-specific characteristics seem to define different modes of action for compound **19**’s action, as summarized in Fig. 7. An in vivo i.p. dosage of only 5 mg/kg significantly reduced the 4T1 breast tumor growth in BALB/c mice. Moreover, a further increase of the compound’s dosage (20 mg/kg) did not show additional improvement of the antitumor effect, indicating that the therapeutically effective dose was already reached with 5 mg/kg (or below). The impact of the pattern of the side chains attached to the 9-azaglycophymine core on the biological activity, i.e., a rudimentary structure–activity relationship, was studied and several structural features were found to be essential for activity. Neither the absence of substitution nor the presence of electron-donating substitution resulted in cytotoxic derivatives within the scope of this study. However, introduction of an electron-withdrawing chloro or especially a nitro group renders the 9-azaglycophymine derivatives cytotoxic, interestingly without a clear relevance of the positioning of the nitro group. This opens the door to substitute the nitro group by other, metabolically better suited (more stable) electron withdrawing substituents and to produce more active and selective compounds through medicinal chemistry optimization. Compound **19**, harboring it at the para (4'-) position of the phenyl group, showed the lowest IC_50_ value. Based on the positive results, further structural optimizations of 9-aza-glycophymine derivatives, ideally avoiding the potential deficits that aromatic nitro compounds can (but not necessarily) have in vivo, deserve further evaluation. As a conclusion, the study highlights that azaglycophymines, in light of these results, are very promising for advanced drug R&D in future studies.

## Supplementary Information

Supplementary Information.

## Data Availability

All data underlying the analyses and findings of this study are available from the corresponding author upon reasonable request.

## References

[1] Lambert, A. W., Pattabiraman, D. R. & Weinberg, R. A. Emerging biological principles of metastasis. *Cell* **168**(4), 670–691. 10.1016/j.cell.2016.11.037 (2017). 28187288 10.1016/j.cell.2016.11.037 PMC5308465

[2] Bray, F. *et al.* Global cancer statistics 2022: GLOBOCAN estimates of incidence and mortality worldwide for 36 cancers in 185 countries. *CA Cancer J. Clin.* **74**(3), 229–263. 10.3322/caac.21834 (2024). 38572751 10.3322/caac.21834

[3] Orrantia-Borunda, E., Anchondo-Nuñez, P., Acuña-Aguilar, L. E., Gómez-Valles, F. O. & Ramírez-Valdespino, C. A. Subtypes of breast cancer. In *Breast Cancer *(eds Mayrovitz, H. N.) (Exon Publications, 2022, accessed 14 December 2023); http://www.ncbi.nlm.nih.gov/books/NBK583808/36122153

[4] Ahmad, I. Tamoxifen a pioneering drug: An update on the therapeutic potential of tamoxifen derivatives. *Eur. J. Med. Chem.* **143**, 515–531. 10.1016/j.ejmech.2017.11.056 (2018). 29207335 10.1016/j.ejmech.2017.11.056

[5] Bobach, C. *et al.* Multiple readout assay for hormonal (androgenic and antiandrogenic) and cytotoxic activity of plant and fungal extracts based on differential prostate cancer cell line behavior. *J. Ethnopharmacol.* **155**(1), 721–730. 10.1016/j.jep.2014.06.008 (2014). 24945396 10.1016/j.jep.2014.06.008

[6] Foglietta, J. *et al.* Cardiotoxicity of aromatase inhibitors in breast cancer patients. *Clin. Breast Cancer* **17**(1), 11–17. 10.1016/j.clbc.2016.07.003 (2017). 27561703 10.1016/j.clbc.2016.07.003

[7] Diaby, V. *et al.* A cost-effectiveness analysis of trastuzumab-containing treatment sequences for HER-2 positive metastatic breast cancer patients in Taiwan. *Breast (Edinburgh, Scotland)* **49**, 141–148. 10.1016/j.breast.2019.11.012 (2020). 31805500 10.1016/j.breast.2019.11.012 PMC7375554

[8] McKeage, K. & Perry, C. M. Trastuzumab: A review of its use in the treatment of metastatic breast cancer overexpressing HER2. *Drugs* **62**(1), 209–243. 10.2165/00003495-200262010-00008 (2002). 11790161 10.2165/00003495-200262010-00008

[9] Lyons, T. G. Targeted therapies for triple-negative breast cancer. *Curr. Treat. Options Oncol.* **20**(11), 82. 10.1007/s11864-019-0682-x (2019). 31754897 10.1007/s11864-019-0682-x

[10] Heimes, A.-S. & Schmidt, M. Atezolizumab for the treatment of triple-negative breast cancer. *Expert Opin. Investig. Drugs* **28**(1), 1–5. 10.1080/13543784.2019.1552255 (2019). 30474425 10.1080/13543784.2019.1552255

[11] Hsu, J.-Y., Chang, C.-J. & Cheng, J.-S. Survival, treatment regimens and medical costs of women newly diagnosed with metastatic triple-negative breast cancer. *Sci. Rep.*10.1038/s41598-021-04316-2 (2022). 35031634 10.1038/s41598-021-04316-2 PMC8760241

[12] Tobe, M. *et al.* Discovery of quinazolines as a novel structural class of potent inhibitors of NF-kappa B activation. *Bioorganic Med. Chem.* **11**(3), 383–391. 10.1016/s0968-0896(02)00440-6 (2003). 10.1016/s0968-0896(02)00440-6 12517433

[13] Guillon, R. *et al.* Discovery of a novel broad-spectrum antifungal agent derived from albaconazole. *ACS Med. Chem. Lett.* **4**(2), 288–292. 10.1021/ml300429p (2013). 24900660 10.1021/ml300429p PMC4027234

[14] Jiang, S. *et al.* Antimalarial activities and therapeutic properties of febrifugine analogs. *Antimicrob. Agents Chemother.* **49**(3), 1169–1176. 10.1128/AAC.49.3.1169-1176.2005 (2005). 15728920 10.1128/AAC.49.3.1169-1176.2005 PMC549280

[15] Sim, E. H., Yang, I. A., Wood-Baker, R., Bowman, R. V. & Fong, K. M. Gefitinib for advanced non-small cell lung cancer. *Cochrane Database Syst. Rev.*10.1002/14651858.CD006847.pub2 (2018). 29336009 10.1002/14651858.CD006847.pub2 PMC6491254

[16] Wang, Y., Schmid-Bindert, G. & Zhou, C. Erlotinib in the treatment of advanced non-small cell lung cancer: An update for clinicians. *Ther. Adv. Med. Oncol.* **4**(1), 19–29. 10.1177/1758834011427927 (2012). 22229045 10.1177/1758834011427927 PMC3244201

[17] Yoshikawa, D. *et al.* Vandetanib (ZD6474), an inhibitor of VEGFR and EGFR signalling, as a novel molecular-targeted therapy against cholangiocarcinoma. *Br. J. Cancer* **100**(8), 1257–1266. 10.1038/sj.bjc.6604988 (2009). 19319137 10.1038/sj.bjc.6604988 PMC2676540

[18] Blackledge, G. New developments in cancer treatment with the novel thymidylate synthase inhibitor raltitrexed (‘Tomudex’). *Br. J. Cancer* **77**(Suppl 2), 29–37. 10.1038/bjc.1998.423 (1998). 9579853 10.1038/bjc.1998.423 PMC2149719

[19] Ahmad, I. An insight into the therapeutic potential of quinazoline derivatives as anticancer agents. *MedChemComm* **8**(5), 871–885. 10.1039/c7md00097a (2017). 30108803 10.1039/c7md00097a PMC6072504

[20] Pakrashi, S. C., Bhattacharyya, J., Johnson, L. F. & Budzikiewicz, H. Studies on indian medicinal plants—VI: Structures of glycosmicine, glycorine and glycosminine, the minor alkaloids from *Glycosmis**arborea* (roxb.) DC. *Tetrahedron* **19**(6), 1011–1026. 10.1016/S0040-4020(01)99356-1 (1963). 13941148

[21] Wang, C.-J. *et al.* Discovery of penipanoid C-inspired 2-(3,4,5-trimethoxybenzoyl)quinazolin-4(3*H*)-one derivatives as potential anticancer agents by inhibiting cell proliferation and inducing apoptosis in hepatocellular carcinoma cells. *Eur. J. Med. Chem.* **224**, 113671. 10.1016/j.ejmech.2021.113671 (2021). 34237623 10.1016/j.ejmech.2021.113671

[22] Dai, X., Cheng, H., Bai, Z. & Li, J. Breast cancer cell line classification and its relevance with breast tumor subtyping. *J. Cancer* **8**(16), 3131–3141. 10.7150/jca.18457 (2017). 29158785 10.7150/jca.18457 PMC5665029

[23] Riaz, M. *et al.* miRNA expression profiling of 51 human breast cancer cell lines reveals subtype and driver mutation-specific miRNAs. *Breast Cancer Res. BCR* **15**(2), R33. 10.1186/bcr3415 (2013). 23601657 10.1186/bcr3415 PMC3672661

[24] Witt, B. L. & Tollefsbol, T. O. Molecular, cellular, and technical aspects of breast cancer cell lines as a foundational tool in cancer research. *Life*10.3390/life13122311 (2023). 38137912 10.3390/life13122311 PMC10744609

[25] Veß, A. *et al.* A dual phenotype of MDA-MB-468 cancer cells reveals mutual regulation of tensin3 and adhesion plasticity. *J. Cell Sci.* **130**(13), 2172–2184. 10.1242/jcs.200899 (2017). 28515231 10.1242/jcs.200899

[26] Padró, M. *et al.* Genome-independent hypoxic repression of estrogen receptor alpha in breast cancer cells. *BMC Cancer* **17**(1), 203. 10.1186/s12885-017-3140-9 (2017). 28320353 10.1186/s12885-017-3140-9 PMC5358051

[27] Tassone, P. *et al.* BRCA1 expression modulates chemosensitivity of BRCA1-defective HCC1937 human breast cancer cells. *Br. J. Cancer* **88**(8), 1285–1291. 10.1038/sj.bjc.6600859 (2003). 12698198 10.1038/sj.bjc.6600859 PMC2747554

[28] Zhang, Y. *et al.* Long noncoding RNA LINP1 regulates repair of DNA double-strand breaks in triple-negative breast cancer. *Nat. Struct. Mol. Biol.* **23**(6), 522–530. 10.1038/nsmb.3211 (2016). 27111890 10.1038/nsmb.3211 PMC4927085

[29] Luberto, C. *et al.* Inhibition of tumor necrosis factor-induced cell death in MCF7 by a novel inhibitor of neutral sphingomyelinase. *J. Biol. Chem.* **277**(43), 41128–41139. 10.1074/jbc.M206747200 (2002). 12154098 10.1074/jbc.M206747200

[30] Khan, M. F. *et al.* Cichorins D–F: Three new compounds from cichorium intybus and their biological effects. *Molecules (Basel, Switzerland)* **25**(18), E4160. 10.3390/molecules25184160 (2020). 10.3390/molecules25184160 PMC7570803 32932909

[31] Feoktistova, M., Geserick, P. & Leverkus, M. Crystal violet assay for determining viability of cultured cells. *Cold Spring Harb. Protoc.* **2016**(4), pdb.prot087379. 10.1101/pdb.prot087379 (2016). 27037069 10.1101/pdb.prot087379

[32] Morgan, I., Wessjohann, L. A. & Kaluđerović, G. N. In vitro anticancer screening and preliminary mechanistic study of A-ring substituted anthraquinone derivatives. *Cells* **11**(1), 168. 10.3390/cells11010168 (2022). 35011730 10.3390/cells11010168 PMC8750254

[33] Kaluđerović, G. N. *et al.* Ruthenium(II) p-cymene complex bearing 2,2′-dipyridylamine targets caspase 3 deficient MCF-7 breast cancer cells without disruption of antitumor immune response. *J. Inorg. Biochem.* **153**, 315–321. 10.1016/j.jinorgbio.2015.09.006 (2015). 26428537 10.1016/j.jinorgbio.2015.09.006

[34] Krajnović, T., Kaluđerović, G. N., Wessjohann, L. A., Mijatović, S. & Maksimović-Ivanić, D. Versatile antitumor potential of isoxanthohumol: Enhancement of paclitaxel activity in vivo. *Pharmacol. Res.* **105**, 62–73. 10.1016/j.phrs.2016.01.011 (2016). 26784390 10.1016/j.phrs.2016.01.011

[35] Maksimovic-Ivanic, D. *et al.* Anticancer properties of the novel nitric oxide-donating compound (S, R)-3-phenyl-4,5-dihydro-5-isoxazole acetic acid-nitric oxide in vitro and in vivo. *Mol. Cancer Ther.* **7**(3), 510–520. 10.1158/1535-7163.MCT-07-2037 (2008). 18347138 10.1158/1535-7163.MCT-07-2037

[36] Faustino-Rocha, A. *et al.* Estimation of rat mammary tumor volume using caliper and ultrasonography measurements. *Lab Anim.* **42**(6), 217–224. 10.1038/laban.254 (2013). 10.1038/laban.254 23689461

[37] Evangelatov, A. *et al.* Epirubicin loading in poly(butyl cyanoacrylate) nanoparticles manifests via altered intracellular localization and cellular response in cervical carcinoma (HeLa) cells. *Drug Deliv.* **23**(7), 2235–2244. 10.3109/10717544.2014.962117 (2016). 25268149 10.3109/10717544.2014.962117

[38] Murugan, S. & Amaravadi, R. K. Methods for studying autophagy within the tumor microenvironment. *Adv. Exp. Med. Biol.* **899**, 145–166. 10.1007/978-3-319-26666-4_9 (2016). 27325266 10.1007/978-3-319-26666-4_9 PMC5451257

[39] Pervaiz, S. & Clement, M.-V. Tumor intracellular redox status and drug resistance-serendipity or a causal relationship?. *Curr. Pharm. Des.* **10**(16), 1969–1977 (2004). 15180532 10.2174/1381612043384411

[40] Tao, K., Fang, M., Alroy, J. & Sahagian, G. G. Imagable 4T1 model for the study of late stage breast cancer. *BMC Cancer* **8**, 228. 10.1186/1471-2407-8-228 (2008). 18691423 10.1186/1471-2407-8-228 PMC2529338

[41] Hostettmann, K., Dey, P. M. & Harborne, J. B. *Methods in Plant Biochemistry* Vol. 6 (Academic Press, 1991).

[42] Rickert, D. E. Metabolism of nitroaromatic compounds. *Drug Metab. Rev.* **18**(1), 23–53. 10.3109/03602538708998299 (1987). 3311683 10.3109/03602538708998299

[43] Kovacic, P. & Somanathan, R. Nitroaromatic compounds: Environmental toxicity, carcinogenicity, mutagenicity, therapy and mechanism. *J. Appl. Toxicol.* **34**(8), 810–824. 10.1002/jat.2980 (2014). 24532466 10.1002/jat.2980

[44] Ringe, D., Turesky, R. J., Skipper, P. L. & Tannenbaum, S. R. Structure of the single stable hemoglobin adduct formed by 4-aminobiphenyl in vivo. *Chem. Res. Toxicol.* **1**(1), 22–24. 10.1021/tx00001a003 (1988). 2979706 10.1021/tx00001a003

[45] Sabbioni, G. Hemoglobin adducts and urinary metabolites of arylamines and nitroarenes. *Chem. Res. Toxicol.* **30**(10), 1733–1766. 10.1021/acs.chemrestox.7b00111 (2017). 28933159 10.1021/acs.chemrestox.7b00111

[46] Cenas, N., Prast, S., Nivinskas, H., Sarlauskas, J. & Arnér, E. S. J. Interactions of Nitroaromatic compounds with the mammalian selenoprotein thioredoxin reductase and the relation to induction of apoptosis in human cancer cells*. *J. Biol. Chem.* **281**(9), 5593–5603. 10.1074/jbc.M511972200 (2006). 16354662 10.1074/jbc.M511972200

[47] Li, J. & Yuan, J. Caspases in apoptosis and beyond. *Oncogene* **27**(48), 6194–6206. 10.1038/onc.2008.297 (2008). 18931687 10.1038/onc.2008.297

[48] Zhang, J. H. & Xu, M. DNA fragmentation in apoptosis. *Cell Res.* **10**(3), 205–211. 10.1038/sj.cr.7290049 (2000). 11032172 10.1038/sj.cr.7290049

[49] Jänicke, R. U. MCF-7 breast carcinoma cells do not express caspase-3. *Breast Cancer Res. Treat.* **117**(1), 219–221. 10.1007/s10549-008-0217-9 (2009). 18853248 10.1007/s10549-008-0217-9

[50] Dhuriya, Y. K. & Sharma, D. Necroptosis: a regulated inflammatory mode of cell death. *J. Neuroinflammation* **15**(1), 199. 10.1186/s12974-018-1235-0 (2018). 29980212 10.1186/s12974-018-1235-0 PMC6035417

[51] Solovieva, M. *et al.* Disulfiram oxy-derivatives induce entosis or paraptosis-like death in breast cancer MCF-7 cells depending on the duration of treatment. *Biochim. Biophys. Acta BBA Gen. Subj.* **1866**(9), 130184. 10.1016/j.bbagen.2022.130184 (2022). 10.1016/j.bbagen.2022.130184 35660414

[52] Prat, A. *et al.* Characterization of cell lines derived from breast cancers and normal mammary tissues for the study of the intrinsic molecular subtypes. *Breast Cancer Res. Treat.* **142**(2), 237–255. 10.1007/s10549-013-2743-3 (2013). 24162158 10.1007/s10549-013-2743-3 PMC3832776

[53] Mueller, K. L., Yang, Z.-Q., Haddad, R., Ethier, S. P. & Boerner, J. L. EGFR/Met association regulates EGFR TKI resistance in breast cancer. *J. Mol. Signal.* **5**, 8. 10.1186/1750-2187-5-8 (2010). 20624308 10.1186/1750-2187-5-8 PMC2911419

[54] Yamaguchi, H. *et al.* Caspase-independent cell death is involved in the negative effect of EGF receptor inhibitors on cisplatin in non-small cell lung cancer cells. *Clin. Cancer Res. Off. J. Am. Assoc. Cancer Res.* **19**(4), 845–854. 10.1158/1078-0432.CCR-12-2621 (2013). 10.1158/1078-0432.CCR-12-2621 PMC3703145 23344263

[55] Pulaski, B. A. & Ostrand-Rosenberg, S. Mouse 4T1 breast tumor model. *Curr. Protoc. Immunol.*10.1002/0471142735.im2002s39 (2001). 18432775 10.1002/0471142735.im2002s39

[56] Woo, Y., Lee, H.-J., Jung, Y. M. & Jung, Y.-J. Regulated necrotic cell death in alternative tumor therapeutic strategies. *Cells*10.3390/cells9122709 (2020). 33348858 10.3390/cells9122709 PMC7767016

[57] Richards, C. H., Mohammed, Z., Qayyum, T., Horgan, P. G. & McMillan, D. C. The prognostic value of histological tumor necrosis in solid organ malignant disease: A systematic review. *Future Oncol.* **7**(10), 1223–1235. 10.2217/fon.11.99 (2011). 21992733 10.2217/fon.11.99

[58] Dietzel, M. *et al.* The necrosis sign in magnetic resonance-mammography: Diagnostic accuracy in 1,084 histologically verified breast lesions. *Breast J.* **16**(6), 603–608. 10.1111/j.1524-4741.2010.00982.x (2010). 21070437 10.1111/j.1524-4741.2010.00982.x

[59] Zhang, Y. *et al.* Clinicopathological study of centrally necrotizing carcinoma of the breast. *BMC Cancer* **15**(1), 282. 10.1186/s12885-015-1305-y (2015). 25880163 10.1186/s12885-015-1305-y PMC4403997

[60] Pu, R. T., Schott, A. F., Sturtz, D. E., Griffith, K. A. & Kleer, C. G. Pathologic features of breast cancer associated with complete response to neoadjuvant chemotherapy: Importance of tumor necrosis. *Am. J. Surg. Pathol.* **29**(3), 354. 10.1097/01.pas.0000152138.89395.fb (2005). 15725804 10.1097/01.pas.0000152138.89395.fb

