# Supplementary material for: The impact of 9-azaglycophymine and phenylguanidine derivatives on the proliferation of various breast cancer cell lines in vitro and in vivo
Source: Sci Rep. 2024 Nov 15;14:28126. doi: 10.1038/s41598-024-71624-8 (PMC11568214; doi:10.1038/s41598-024-71624-8)
Supplement: Supplementary file 1 — Supplementary Information. [file 41598_2024_71624_MOESM1_ESM.docx]

The impact of 9-azaglycophymine and phenylguanidine derivatives on the proliferation of various breast cancer cell lines *in vitro* and *in vivo*

Ibrahim Morgan^1^, Robert Rennert^1*^, Robert Berger^1,2^, Sanja Jelača^3^, Danijela Maksimović-Ivanić^3^, Duško Dunđerović^4^, Sanja Mijatović^3^, Goran N. Kaluđerović^1,5^ and Ludger A. Wessjohann^1*^

^1^ Department of Bioorganic Chemistry, Leibniz Institute of Plant Biochemistry, Weinberg 3, 06120, Halle (Saale), Germany

^2^ Current address: Berlin, Germany (formerly: Department of Bioorganic Chemistry, Leibniz Institute of Plant Biochemistry, Weinberg 3, 06120, Halle (Saale), Germany)

^3^ Department of Immunology, Institute for Biological Research ”Siniša Stanković” – National Institute of the Republic of Serbia (IBISS), University of Belgrade, Bulevar despota Stefana 142, 11060 Belgrade, Serbia

^4^ Institute of Pathology, School of Medicine, University of Belgrade, Dr Subotića 8, 11000 Belgrade, Serbia

^5^ Department of Engineering and Natural Sciences, University of Applied Sciences Merseburg, Eberhard-Leibnitz-Straße 2, 06217 Merseburg, Germany

***** Correspondence: wessjohann@ipb-halle.de; robert.rennert@ipb-halle.de

**Table S1** The four human breast cancer cell lines are characterized by different expression levels of ER, PR, HER2/neu and BRCA-1

| **Genes / BC type** | **MCF-7** | **MDA-MB-468** | **HCC1937** | **BT-474** |
| --- | --- | --- | --- | --- |
| **HER2/neu** | - | - | - | + |
| **PR** | + | - | - | + |
| **ER** | + | - | - | + |
| **EGFR** | + | ++++ | +++ | + |
| **BRCA-1** | Allelic loss | Allelic loss | Mutated | Allelic loss |
| **BC type** | HR+ | TNBC | TNBC | HER2+ |

**Table S2**  List of all tested compounds **1** – **49**

| **Compound** | **Structure** | **Compound** | **Structure** |
| --- | --- | --- | --- |
| **1** |  | **2** |  |
| **3** |  | **4** |  |
| **5** |  | **6** |  |
| **7** |  | **8** |  |
| **9** |  | **10** |  |
| **11** |  | **12** |  |
| **13** |  | **14** |  |
| **15** |  | **16** |  |
| **17** |  | **18** |  |
| **19** |  | **20** |  |
| **21** |  | **22** |  |
| **23** |  | **24** |  |
| **25** |  | **26** |  |
| **27** |  | **28** |  |
| **29** |  | **30** |  |
| **31** |  | **32** |  |
| **33** |  | **34** |  |
| **35** |  | **36** |  |
| **37** |  | **38** |  |
| **39** |  | **40** |  |
| **41** |  | **42** |  |
| **43** |  | **44** |  |
| **45** |  | **46** |  |
| **47** |  | **48** |  |
| **49** |  |  |  |


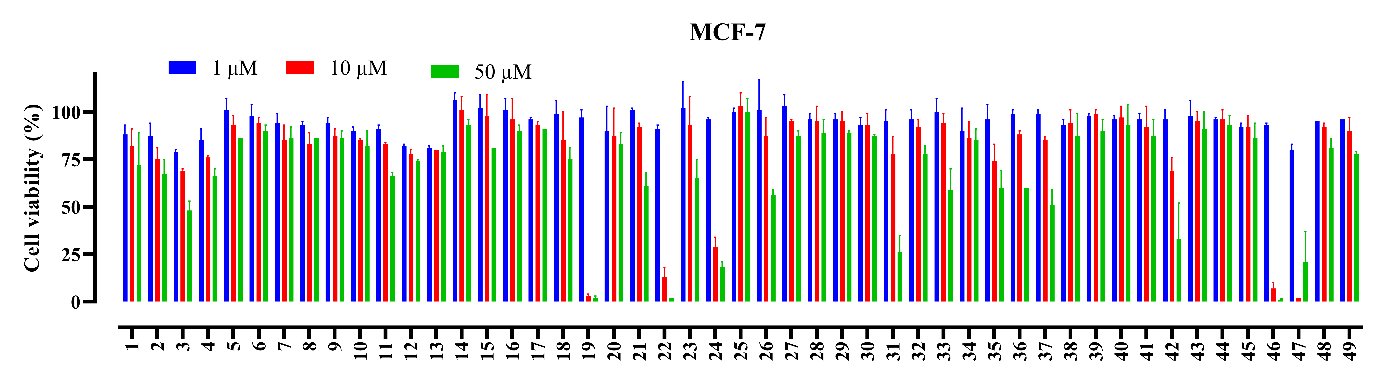

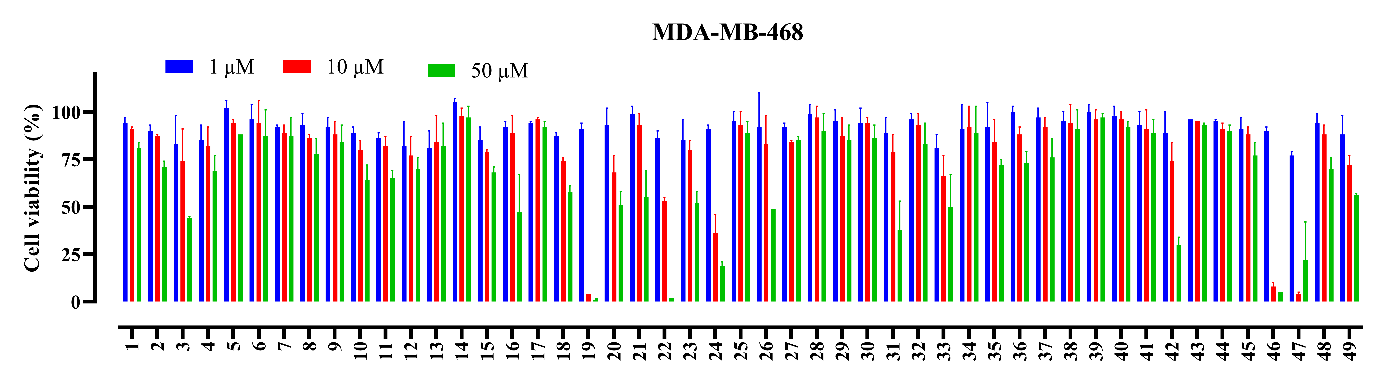
**Fig. S1**  MCF-7 and MDA-MB-468 cell viability after 72 h treatment with the selected compounds and indicated concentrations, viability was determined by using CV assay


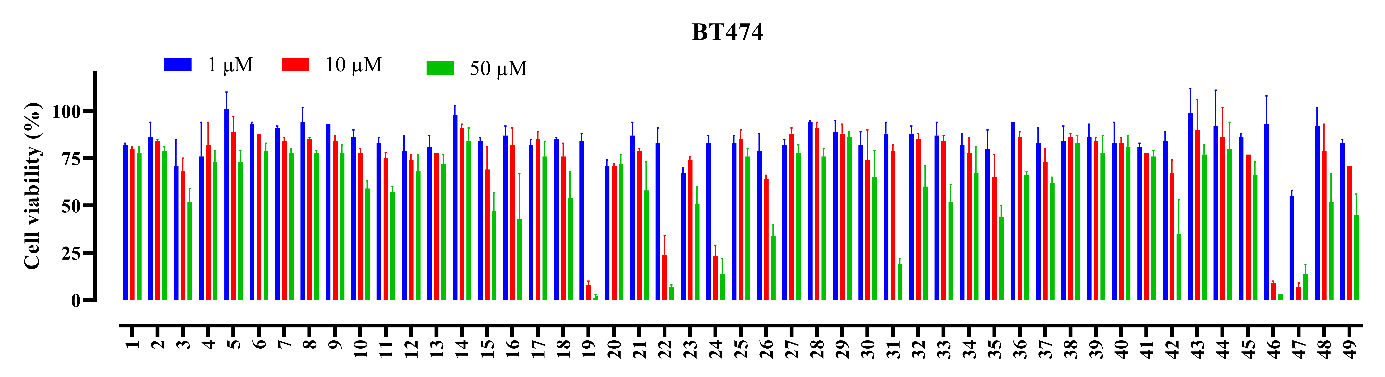


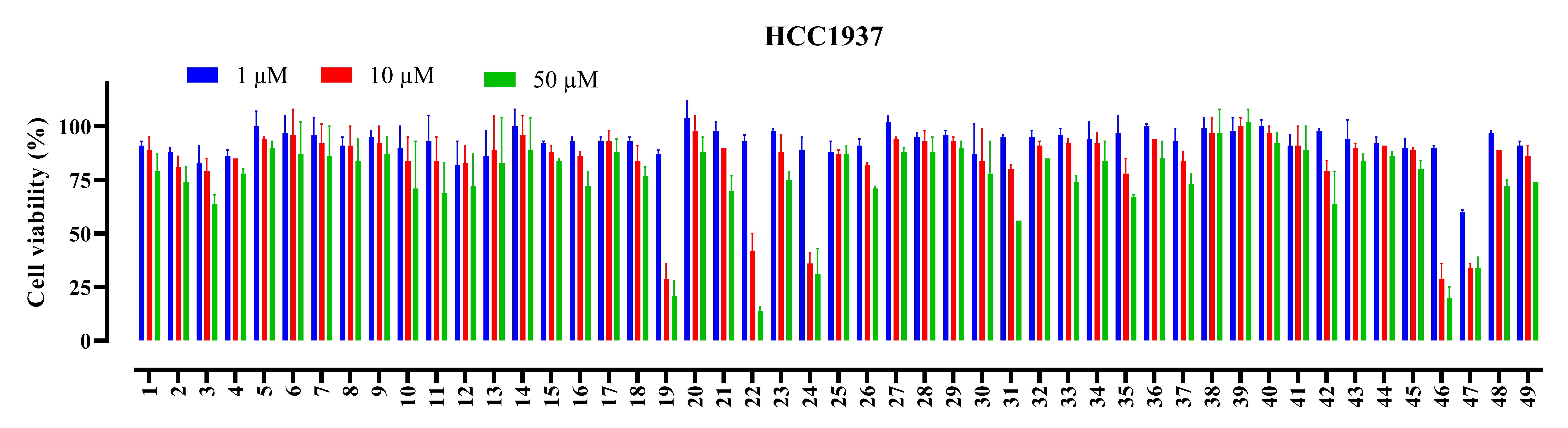


**Fig. S2**  BT-474 and HCC1937 cell viability after 72 h treatment with the selected compounds and indicated concentrations, viability was determined by using CV assay


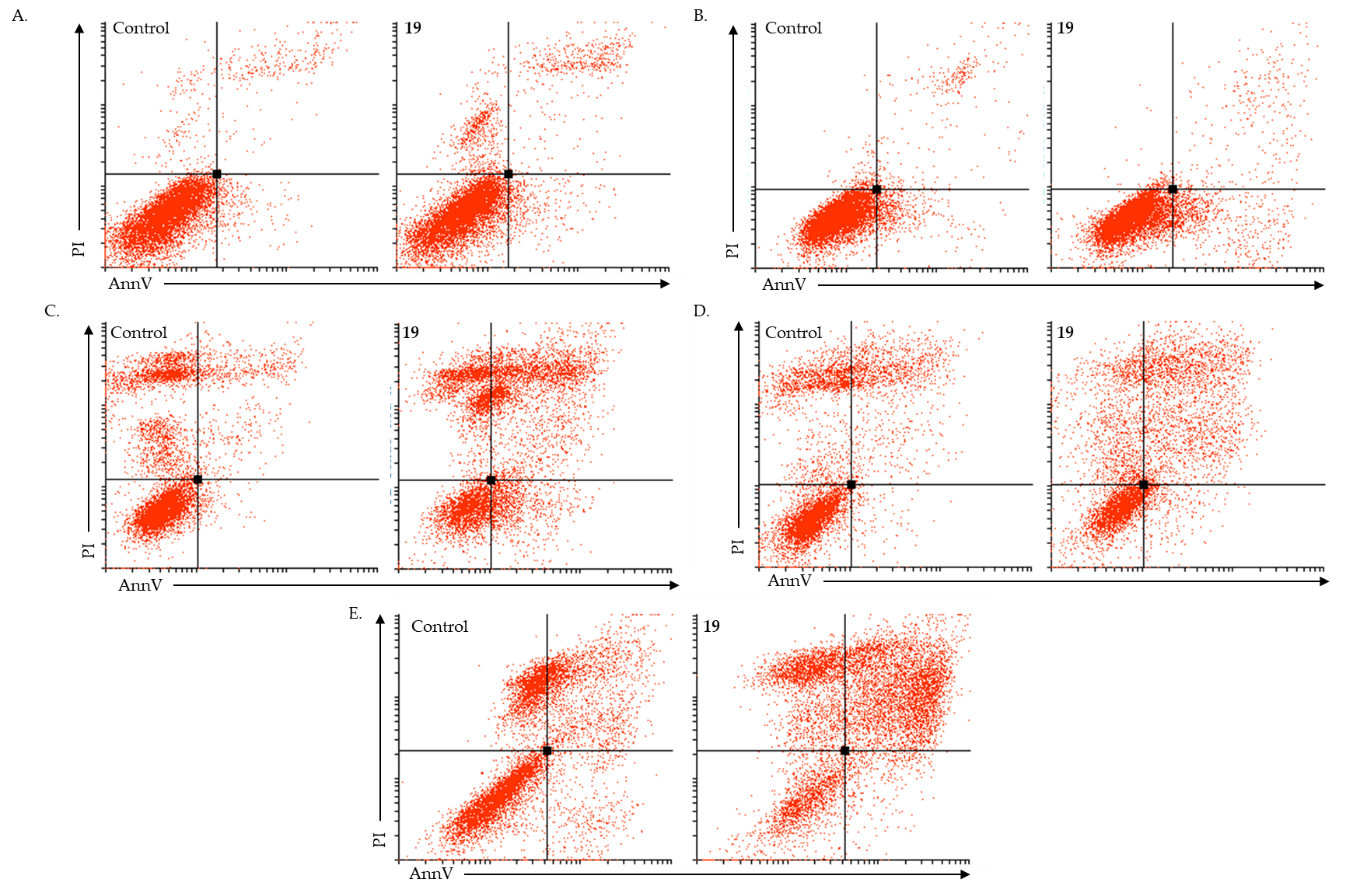


**Fig. S3**  Representative dot plots for the impact of compound **19** applied at IC_50_ for 72 h on the induction of apoptosis, measured by using AnnV/PI double staining against A. MCF-7, B. MDA-MB-468, C. BT-474, D. HCC1937 and E. 4T1. For AnnV stain, the fluorescence intensity was analyzed using excitation at 488 ± 20 nm and emission at 530 ± 30 nm, respectively (plotted at x-axis). For PI dye, the fluorescence intensity was analyzed using excitation and emission at 561 ± 20 and 610 ± 20 nm, respectively (plotted at y-axis). The lower left, lower right and the upper right quadrants represent live cells, early, and late apoptotic cells, respectively


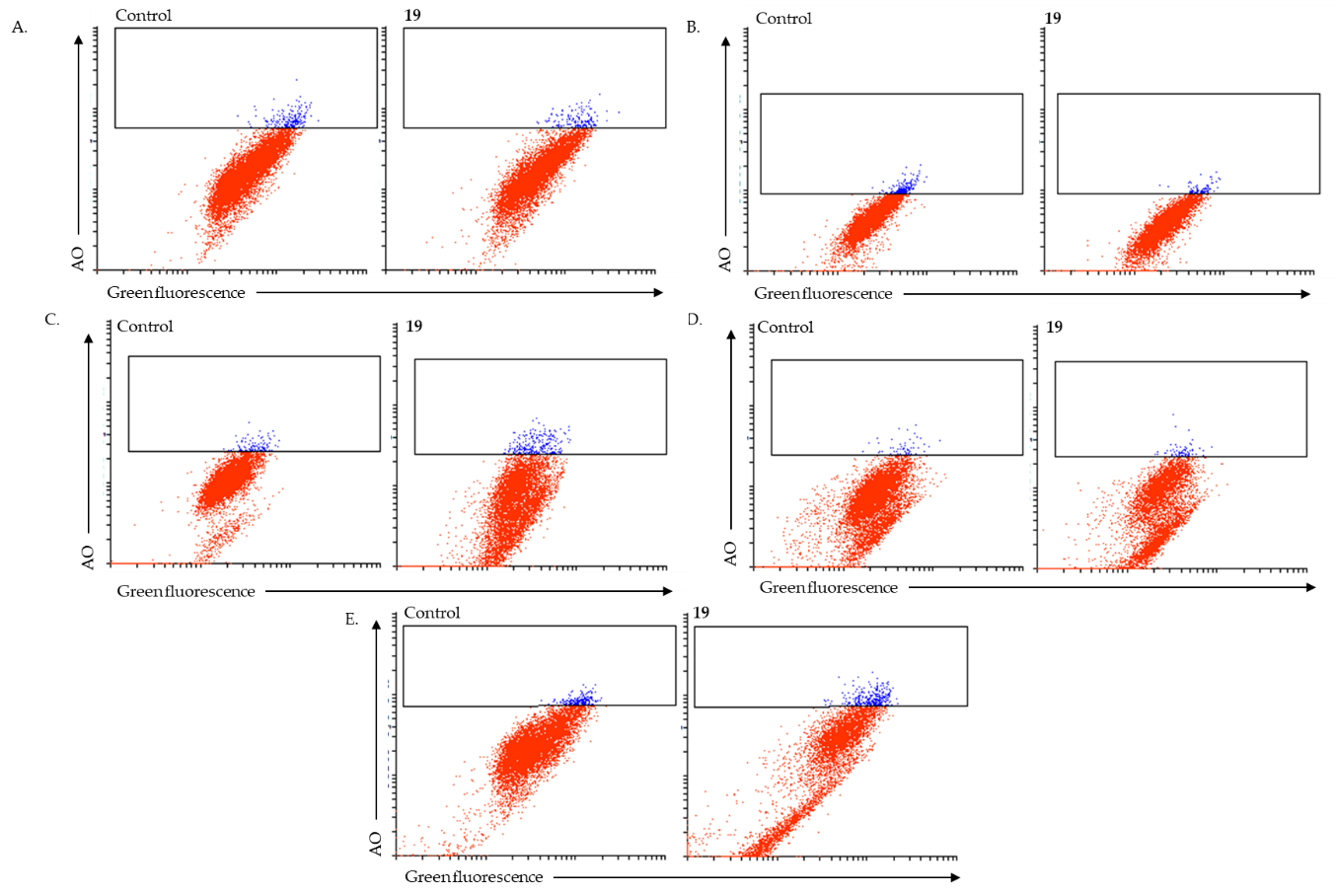


**Fig. S4**  Representative dot plots for the impact of compound **19** applied at IC_50_ for 72 h on the induction of autophagy, measured by performing acridine orange assay: A. MCF-7, B. MDA-MB-468, C. BT-474, D. HCC1937 and E. 4T1. For green fluorescence, the fluorescence intensity was analyzed using excitation at 488 ± 20 nm and emission at 530 ± 30 nm (plotted at x-axis). For AO dye, the fluorescence intensity was analyzed using excitation and emission at 488 ± 20 and 695 ± 40 nm (plotted at y-axis)

**Fig. S5** Representative histograms for the impact of compound **19** applied at IC_50_ for 72 h on the induction of ROS production, determined with DHR assay against A. MCF-7, B. MDA-MB-468, C. BT-474, D. HCC1937 and E. 4T1. For DHR stain, the fluorescence intensity was analyzed using excitation at 488 ± 20 nm and emission at 530 ± 30 nm (plotted at x-axis)


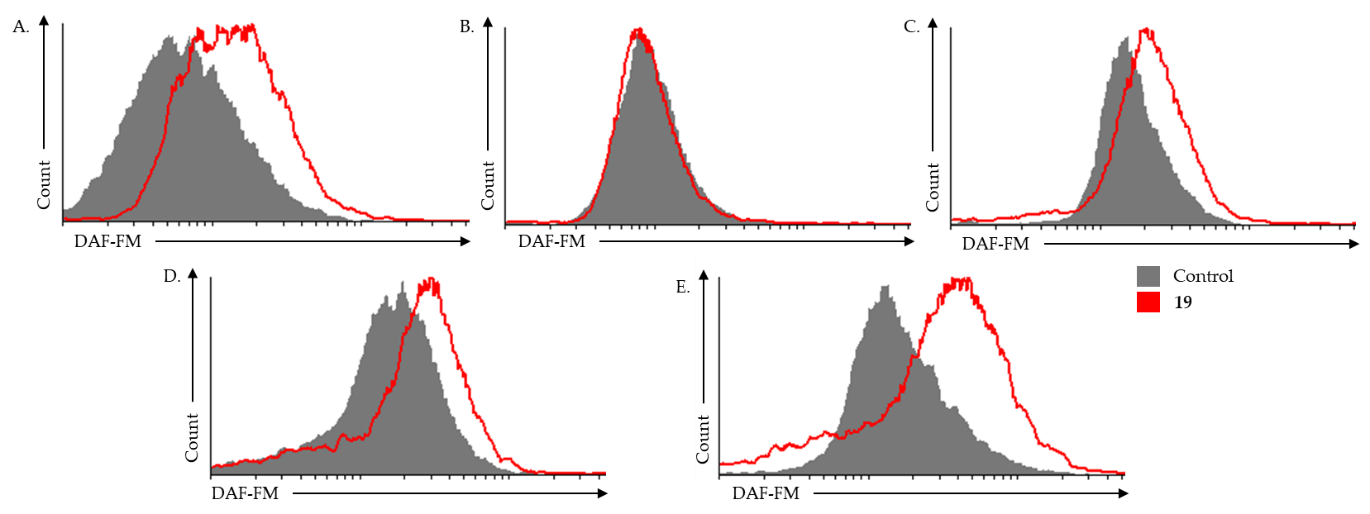


**Fig. S6**  Representative histograms for the impact of compound **19** applied at IC_50_ for 72 h on NO production, measured by using DAF-FM dye: A. MCF-7, B. MDA-MB-468, C. BT-474, D. HCC1937 and E. 4T1. For DAF-FM stain, the fluorescence intensity was analyzed using excitation at 488 ± 20 nm and emission at 530 ± 30 nm (plotted at x-axis)


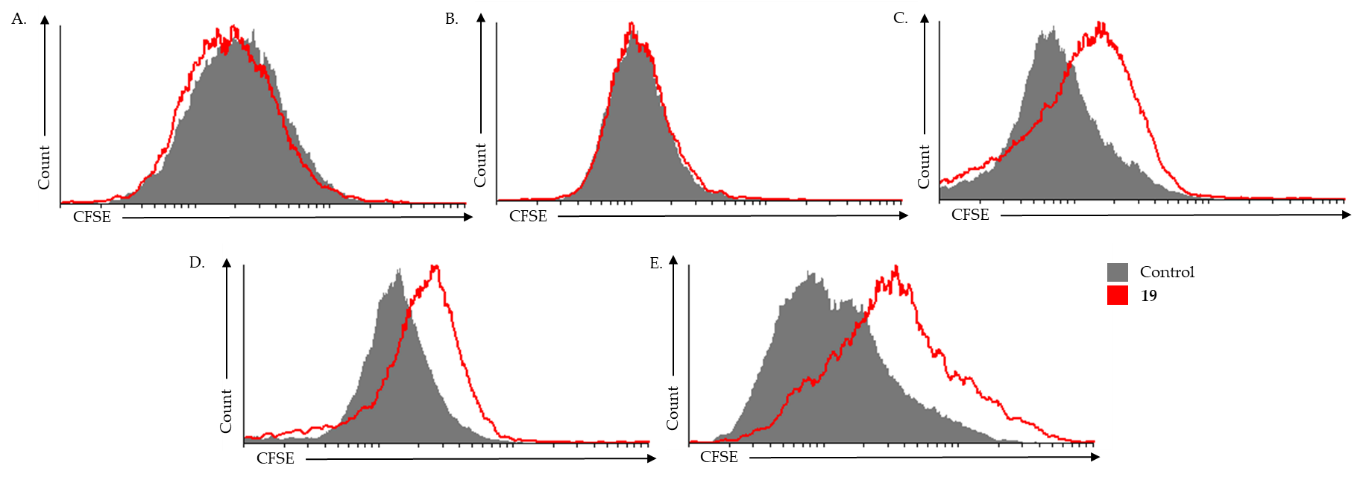


**Fig. S7**  Representative histograms for the impact of compound **19** applied at IC_50_ for 72 h on the inhibition of cells proliferation, in which cells were stained using CFSE reagent, later treated with the lead compound on A. MCF-7, B. MDA-MB-468, C. BT-474, D. HCC1937 and E. 4T1. For CFSE stain, the fluorescence intensity was analyzed using excitation at 488 ± 20 nm and emission at 530 ± 30 nm (plotted at x-axis)


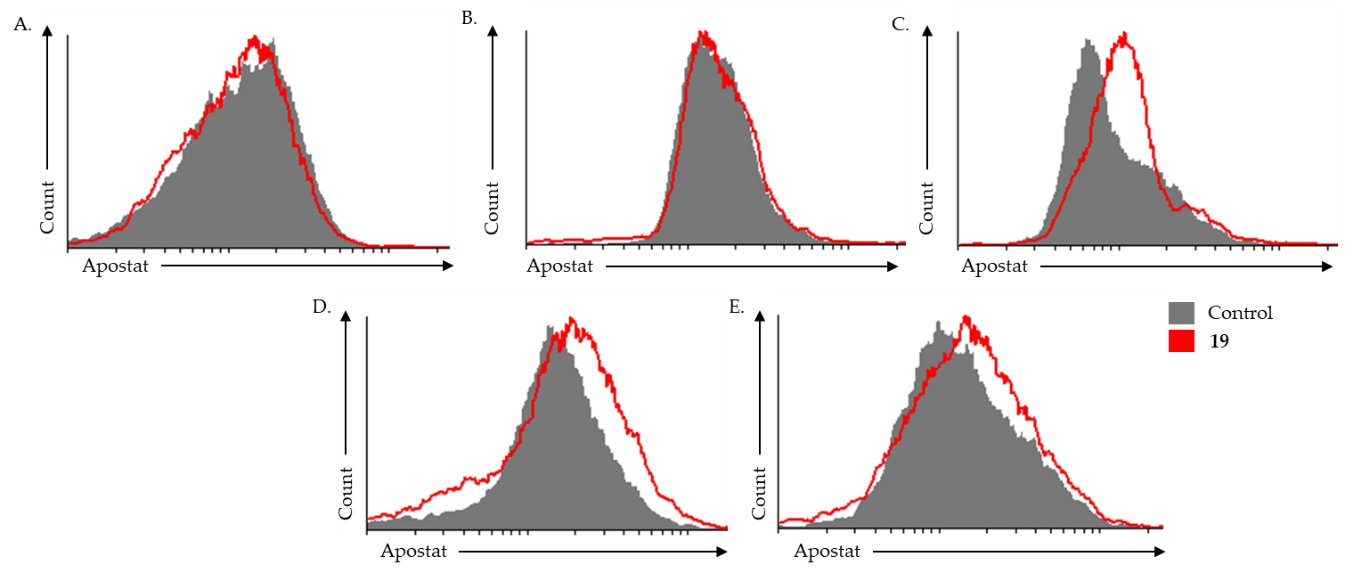


**Fig. S8** Representative histograms for the impact of compound **19** applied at IC_50_ for 72 h on the induction of caspases production, measured by using ApoStat staining kit against A. MCF-7, B. MDA-MB-468, C. BT-474, D. HCC1937 and E. 4T1. For ApoStat stain, the fluorescence intensity was analyzed using excitation at 488 ± 20 nm and emission at 530 ± 30 nm (plotted at x-axis)


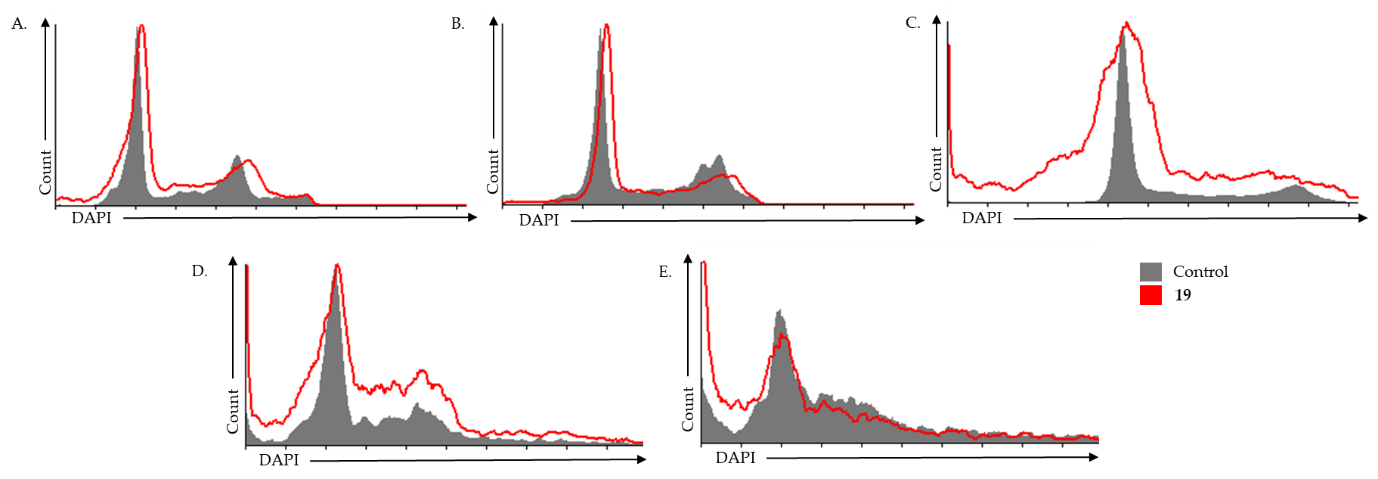


**Fig. S9** Representative histograms for the impact of compound **19** applied at IC_50_ for 72 h on the cell cycle status distribution in G0/G1, S and G2/M phases, measured by using DAPI stain against A. MCF-7, B. MDA-MB-468, C. BT-474, D. HCC1937 and E. 4T1. For DAPI stain, the fluorescence intensity was analyzed using excitation at 375 ± 20 nm and emission at 450 ± 20 nm (plotted at x-axis)


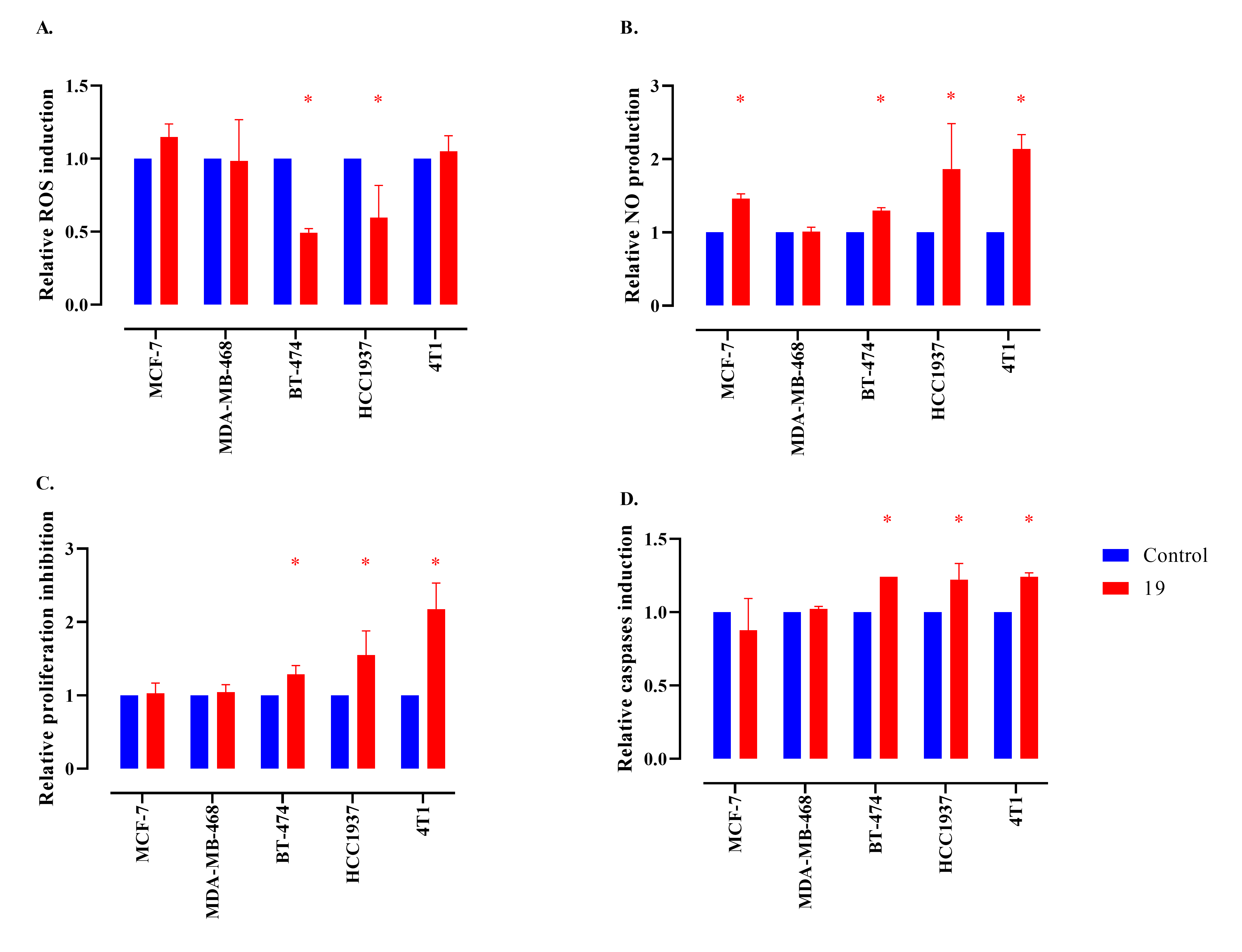


**Fig. S10** Bar graphs representing the impact of compound **19** applied at IC_50_ for 72 h to breast cancer cell lines, on (A) the induction of ROS production, (B) the induction of NO production, (C) the inhibition of cell proliferation, (D) the induction of caspases production. Data were normalized to the corresponding value in the untreated sample. Bars represent the mean values ± standard deviation calculated from three independent measurements. * p < 0.05 compared to the untreated control cells


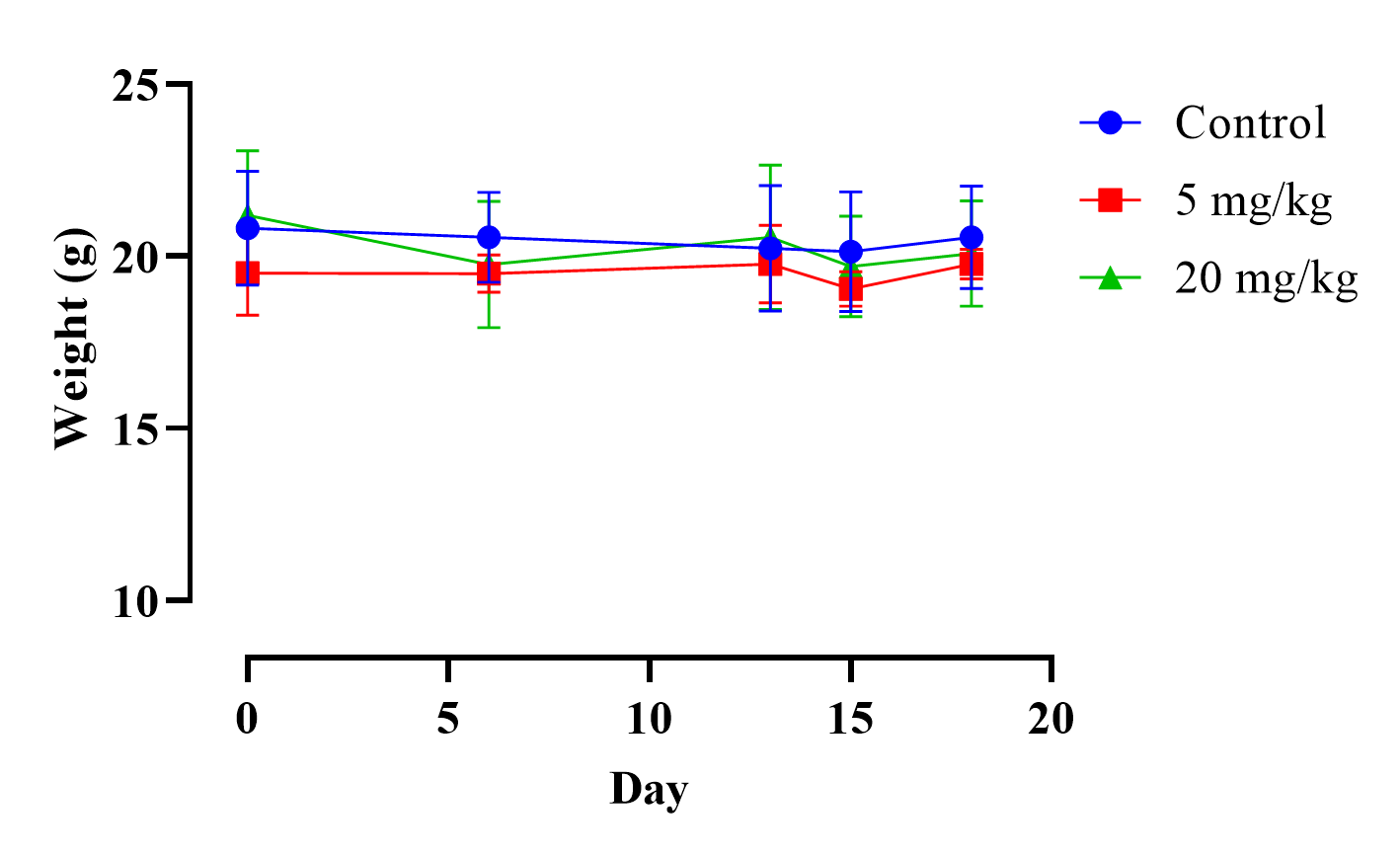


**Fig. S11** Body weights of BALB/c mice upon treatment with compound **19** using 5 and 20 mg/kg. Weight was measured for all mice on day 0, 6, 13, 15 and 18 and represented as a mean value


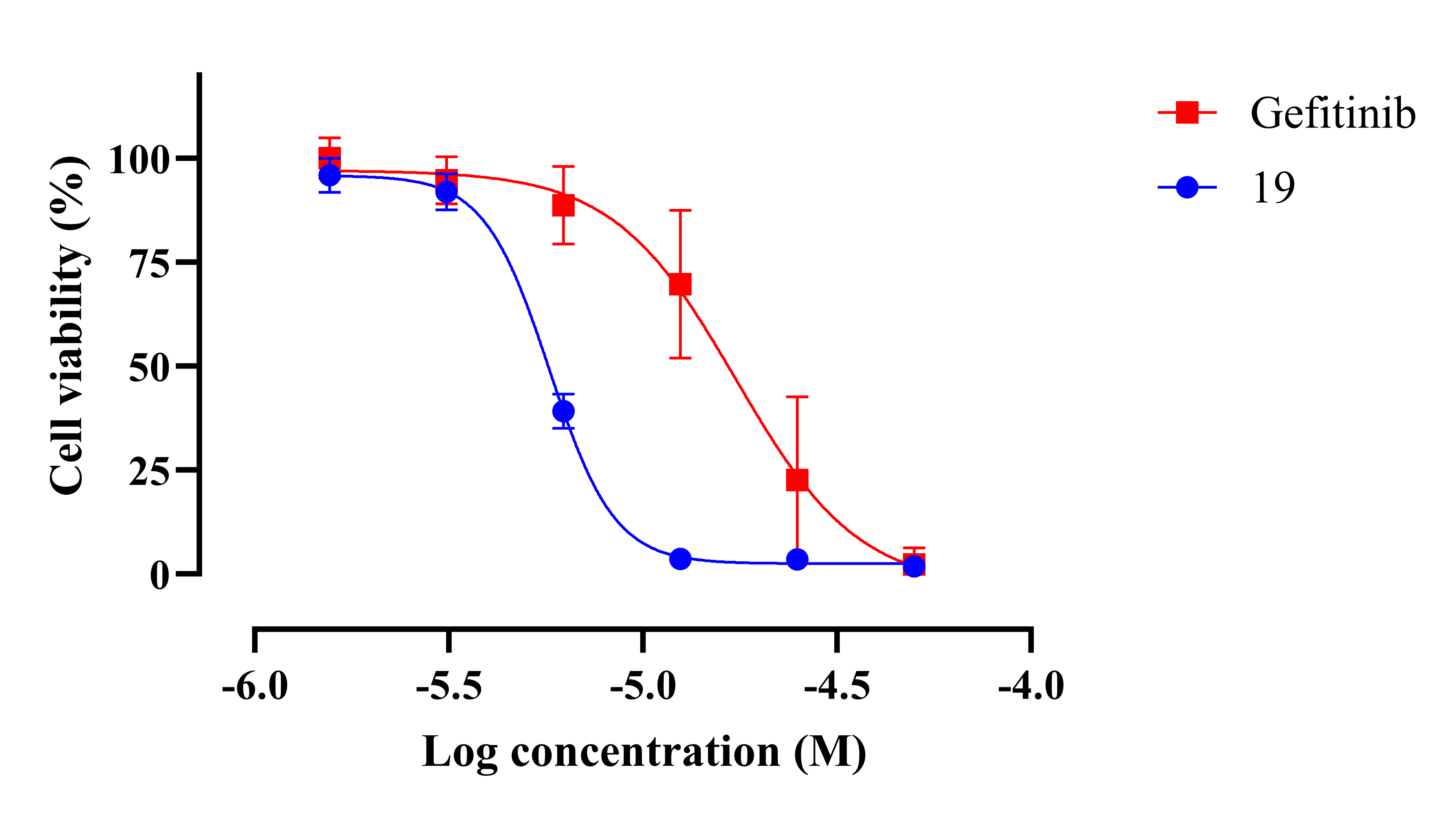


**Fig. S12**  Dose-response curves of the viability of MDA-MB-468 cells after 72 h treatment with the compound **19** and gefitinib, measured by using the CV assay


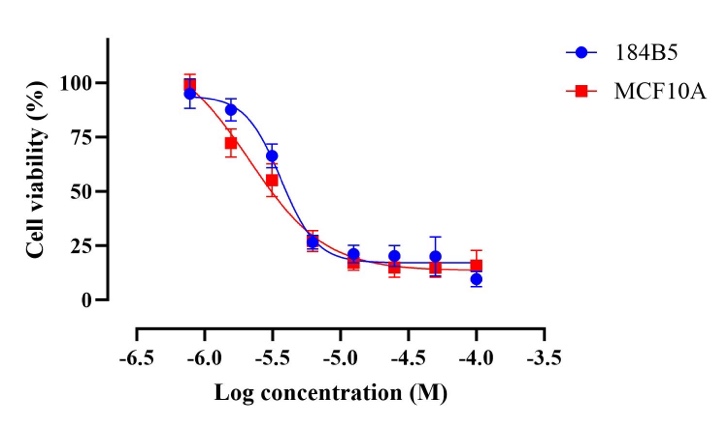


**Fig. S13** Dose-response curves of the viability of 184B5 and MCF10A cells after 72 h treatment with the compound **19**, measured by using the CV assay

**Table S3** Analysis of several biochemical markers in the urine obtained from three mice representing each group. Samples were obtained at day 6 and 20, respectively, representing the first and last day of treatment. The urine analysis was performed using Multistix urine test stripes. “-“: marker was not detectable in the respective urine sample; “+”: marker was detected

| **Biochemical markers** | **Control** | | **5 mg/kg** | | **20 mg/kg** | |
| --- | --- | --- | --- | --- | --- | --- |
|  | Day 6 | Day 20 | Day 6 | Day 20 | Day 6 | Day 20 |
| **Blood** | - | - | - | - | - | - |
|  | - | - | - | - | - | - |
|  | - | - | - | - | - | - |
| **Bilirubin** | moderate | small | small | small | small | small |
|  | moderate | small | small | small | small | small |
|  | moderate | small | small | small | small | moderate |
| **Uroglobin (mg/dL)** | 4 | 8 | 1 | 4 | 4 | 4 |
|  | 8 | 8 | 4 | 4 | 4 | 4 |
|  | 4 | 8 | 4 | 1 | 4 | 8 |
| **Ketones (mg/dL)** | 5 | 10 | 10 | 10 | 10 | 10 |
|  | 10 | 5 | 10 | 10 | 10 | 5 |
|  | 5 | 10 | 10 | 10 | 10 | 10 |
| **Proteins (mg/dL)** | 100 | 100 | 100 | 100 | 30 | 30 |
|  | 100 | 100 | 100 | 100 | 30 | 100 |
|  | 100 | 100 | 30 | 100 | 30 | 100 |
| **Nitriles** | + | - | + | - | + | - |
|  | + | - | - | - | + | - |
|  | + | - | + | - | + | - |
| **Glucose (mg/dL)** | - | 500 | 100 | - | 100 | 250 |
|  | - | - | - | - | 100 | - |
|  | 250 | - | 100 | - | - | 500 |
| **pH** | 6 | 6 | 6 | 6 | 6 | 5 |
|  | 5 | 5 | 5 | 6 | 5 | 6 |
|  | 6 | 6 | 5 | 6 | 5 | 5 |
| **Specific gravity** | 1.025 | 1.025 | 1.025 | 1.030 | 1.025 | 1.030 |
|  | 1.030 | 1.030 | 1.000 | 1.030 | 1.025 | 1.030 |
|  | 1.030 | 1.030 | 1.030 | 1.025 | 1.030 | 1.025 |
| **Leucocytes (cells/µL)** | 75 | 75 | 25 | 25 | 25 | 25 |
|  | 75 | 25 | 25 | 25 | 25 | 25 |
|  | 25 | 25 | 25 | 25 | 25 | 75 |
